# AP1 is a pioneer transcription factor that programmes cell fate through MADS-domain protein tetramerisation

**DOI:** 10.1186/s13059-025-03884-0

**Published:** 2025-12-09

**Authors:** Xiaocai Xu, Manuel Neumann, Frederic Carew, Peilin Chen, Caroline Braeuning, Chloe Zubieta, Jose M. Muino, Cezary Smaczniak, Kerstin Kaufmann

**Affiliations:** 1 Plant Cell and Molecular Biology, Institute of Biology, Humboldt-Universität Zu Berlin, Berlin, Germany; 2 Genomics Platform, Max Delbrück Center for Molecular Medicine in the Helmholtz Association/Berlin Institute of Health, Berlin, Germany; 3 Laboratoire de Physiologie Cellulaire Et Végétale, Univ. Grenoble Alpes, CNRS, CEA, INRAE, IRIG-DBSCI-LPCV, 17 Avenue Des Martyrs, Grenoble, 38054 France

**Keywords:** Plant cell fate programming, Pioneer transcription factors, MADS-domain proteins, Tetramerisation

## Abstract

**Background:**

In animals, pioneer transcription factors (TFs) have long been known to be crucial molecular players in programming cell fate. However, in plants much less is known about this functional class of TFs and how they mechanistically alter local chromatin architecture in order to reprogramme gene regulation to orchestrate cell fate changes.

**Results:**

Here, we provide evidence that APETALA1 (AP1) functions as a pioneer TF in *Arabidopsis thaliana*, facilitated by tetramerisation. Using an integrated combination of multi-omics and high-resolution imaging approaches on both wild-type and AP1 mutant transgenic plants, we show that tetramerisation assists AP1 in providing access to, and enhancing the binding of AP1 to, closed chromatin in vivo*.* This, in turn, allows for a switching of the chromatin state from closed to open to ensure access to target DNA sequences needed for organ specification.

**Conclusions:**

These novel insights provide a mechanistic basis for how AP1 functions as a pioneer factor to reprogramme stem cells towards a “floral ground state”, increasing our understanding of how MADS-domain TFs function as combinatorial units during early *Arabidopsis thaliana* reproductive development.

**Supplementary Information:**

The online version contains supplementary material available at 10.1186/s13059-025-03884-0.

## Background

Apical meristems produce different types of lateral organs during postembryonic plant growth. Re-programming of meristematic identities requires the interplay between TFs, epigenetic regulators, and intercellular signalling. In the reproductive phase, specification of floral meristem identity is mediated by the AP1 and LEAFY (LFY) TFs that activate the expression of floral homeotic genes and control floral organ initiation in concert with other factors [1]. The morphogenesis of flowers is associated with dynamic changes in the chromatin accessibility of cis-regulatory regions, resulting in tissue and stage-specific activation of gene activities. LFY was identified as a pioneer TF that triggers chromatin opening [2, 3]. Genomic analyses suggest that AP1 can also bind to closed chromatin and facilitate an increase in accessibility of previously closed regulatory regions [4].

AP1 is a member of the MIKC class of MADS-domain TFs that have acquired various important functions in developmental phase transitions and organ specification in plants during evolution. These TFs, termed “MIKC-type”, are characterised by a modular protein domain architecture consisting of the DNA-binding MADS domain, followed by an Intervening (I)-domain, the Keratin-like (K) domain that facilitates tetramerisation and a divergent, largely disordered C-terminal domain [5]. MADS-domain proteins bind to their DNA-binding sites called CArG box (general consensus: CC[W]_5–7_GG) as dimers. Tetramerisation of MADS-domain proteins can facilitate simultaneous binding to two CArG boxes at short distances (~ 40–60 bp; [6]) and longer distances [7], resulting in DNA loop formation and thereby potentially influencing the spatial positioning and accessibility of other cis-regulatory elements in promoters of their target genes.

MIKC-type TFs evolved a complex intrafamily protein interaction network, facilitated by the origin of the K-domain in a most recent common ancestor of extant land plants [8, 9]. However, we still do not understand the mechanistic role of tetramerisation of these TFs in mediating developmental phase transitions and organ specification. In this work, we show that tetramerisation is required for effective recognition of DNA-binding sites by AP1 in an in vivo chromatin context and for programming of cell identities in the developing flower.

## Results

### Tetramerisation is required for AP1 function in vivo

**Fig. 1:**
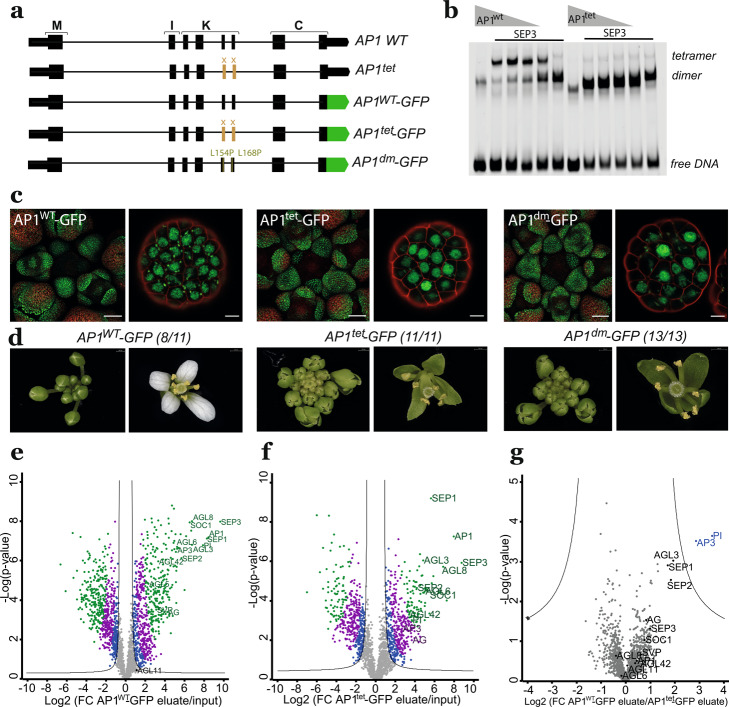
Tetramerisation-deficient AP1 does not complement the *ap1-11* loss-of-function mutant. **a** Schematic representation of gene structures of the *AP1* wildtype locus, the AP1^tet^ CRISPR mutant, and GFP-tagged AP1^WT^, AP1^tet^ and AP1^dm^ expressed from the pAP1 promoter in the *ap1-11* mutant. **b** Electrophoretic Mobility Shift Assay (EMSA) showing the nearly complete loss of heterotetramerisation of AP1^tet^ with SEP3 compared to AP1^WT^ with SEP3, using a DNA probe from the *SEP3* distal enhancer region containing one CArG box (see also Additional file 1: Fig. S1). **c** Confocal images showing expression pattern and sub-cellular localisation of GFP-fused AP1 variants. Intensities were adjusted for individual images. Scale bar 50 µm (images covering different flower meristem stages) or 5 µm (focused images).** d** Representative images of the phenotypes of complementation lines of AP1^WT^-GFP (left), AP1^tet^-GFP (middle), and AP1^dm^-GFP (right) in the *ap1-11* background (see Fig. S3 for *ap1*-*11* and *ap1*^*tet*^ images). Number of transgenic T1 lines showing the representative phenotype out of all analysed T1 lines are indicated by the numbers. **e**–**g** Volcano plots quantifying AP1 complex partners based on IP-MS for inflorescences expressing AP1^WT^-GFP or AP1^tet^-GFP; f) as well as a direct comparison of AP1^WT^-GFP and AP1^tet^-GFP eluates (g). Abbreviated names for all identified MADS-domain proteins are shown. Colour coding: grey or black dots—not significant (FDR < 0.05), blue dots—FDR 0.05, violet dots FDR 0.01, green dots FDR 0.001; the black line is at FDR 0.05. See Additional file 2: Table S1 for data used to plot Fig. 1e–g

Structural and biochemical characterisation of the K-domain of SEPALLATA3 (SEP3) revealed a critical role for an amphipathic α-helix in mediating tetramerisation. Deletions in this α-helix in SEP3 resulted in a reduced capacity to tetramerise accompanied by loss of floral meristem determinacy and partial defects in floral organ development in mutant complementation experiments. However, previous results were inconclusive towards elucidating the mechanistic role of tetramer formation in the function of MADS domain proteins due to residual tetramerisation capacity of the mutant protein [10], or potential compensatory effects by complex partners of SEP3 [11]. In order to address this, we mutagenised the tetramerisation interface of the AP1 protein *in planta* by deleting amino acids 144 to 172 in the K-domain encoded by exons 5 and 6 of the *AP1* genomic locus using Cas9-based exon deletion, resulting in an in-frame fusion of exons 4 and 7 in the endogenous *AP1* genomic locus (*ap1*^*tet*^) (Fig. 1a). In a second approach, we generated complementation lines using an exon 5,6-deleted AP1 mutant fused to GFP in the *ap1*-*11* mutant (AP1^tet^-GFP), and by site-specific mutagenesis of L154P and L168P in the K-domain of a pAP1:AP1-GFP construct transformed into the *ap1*-*11* mutant (AP1^dm^-GFP) (Fig. 1a). Both deletion and amino acid substitutions were predicted to result in a complete loss of tetramerisation based on information from the SEP3 K-domain crystal structure. We confirmed loss of formation of DNA-binding heterotetramers of the AP1 mutant proteins with the partner protein SEP3 by in vitro Electrophoretic Mobility Shift Assays (EMSAs) (Fig. 1b, Additional file 1: Fig. S1b-d, Additional file 1: Fig. S2a), and confirmed in vivo protein expression by Western blot (Additional file 1: Fig. S1a). Confocal imaging of the AP1^WT^-GFP, AP1^tet^-GFP, and AP1^dm^-GFP fusions expressed from the endogenous *AP1* promoter in the *ap1-11* background revealed similar spatiotemporal expression patterns and nuclear localisation (Fig. 1c). Phenotypic analysis of all AP1 tetramerisation-deficient mutant lines (*ap1*^*tet*^, pAP1::AP1^tet^-GFP *ap1*-*11* and pAP1::AP1^dm^-GFP *ap1*-*11*) revealed a phenotype resembling the *ap1-11* mutant with missing petals and sepals transformed into cauline leaf-like organs (Fig. 1c; Additional file 1: Fig. S3). Progression through the early floral meristematic stages was delayed in young inflorescences (Fig. 1c, d Additional file 1: Fig. S3), similar to the *ap1-11* mutant (Additional file 1: Fig. S3). These data suggest that higher-order complex formation is essential for the function of AP1 in petal initiation and sepal specification.

To analyse the tetramerisation capacity of AP1 wildtype and mutant proteins in vivo, we performed IP-MS experiments with AP1^WT^-GFP and AP1^tet^-GFP inflorescences (Additional file 2: Table S1). The data suggest that several MADS-domain proteins can interact with AP1 as part of tetramers and dimers, including SEP proteins, as well as SHORT VEGETATIVE PHASE (SVP) and SUPPRESSOR OF OVEREXPRESSION OF CONSTANS 1 (SOC1), consolidating and extending previous results [12]. Quantitative analysis revealed a reduction of the two homeotic B-class proteins APETALA3 (AP3) and PISTILLATA (PI) in the AP1^tet^-GFP IP-MS sample (Fig. 1e-g). Since AP3 and PI only interact with AP1 as part of a tetrameric complex, this further confirms the decrease of tetramerisation capacity of the AP1^tet^ protein. We identified additional MADS domain interaction partners of AP1 in the complex isolation experiments (Fig. 1e), most of which can form both heterotetramers and heterodimers with AP1 in vitro (Additional file 1: Fig. S1e). In addition, we observed a reduced enrichment of several types of nucleosome remodelling proteins such as BRAHMA (BRM) in the AP1^tet^-GFP compared to AP1^WT^-GFP IP-MS data. Although this reduction was not statistically significant, this finding potentially sheds light on the potential role of AP1 tetramers in the recruitment of chromatin remodellers (Additional file 1: Fig. S4, Additional file 2: Table S1). Together, the results show that tetramerisation is important to mediate the function of AP1 in floral meristem development and organ differentiation.

### Tetramerisation facilitates effective DNA-binding of AP1

**Fig. 2:**
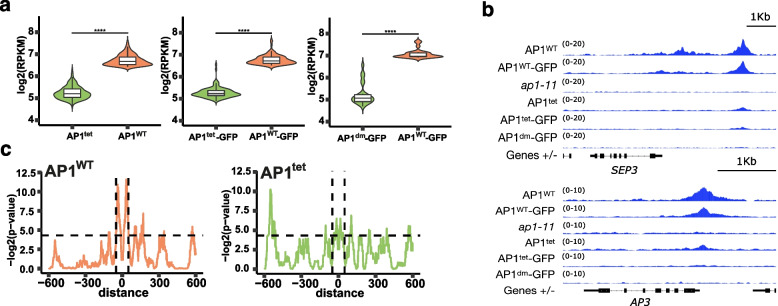
AP1 tetramerisation affects global DNA binding levels in vivo. **a** Number of reads mapped to AP1^WT^ vs. AP1^tet^ ChIP-seq peaks. Significance was tested using the paired two-sided Mann–Whitney test. The boxes extend from the lower to upper quartile values of the data, with a line at the median. The whiskers extend to 1.5 of the interquartile range. **b** Genome browser tracks of ChIP-seq peaks in the promoters of *SEP3* (top) and *AP3* (bottom). The *ap1-11* mutant is used to show one of the background datasets used for peak calling. **c** Enrichment of AP1 SELEX-seq peak distances within the top 388 AP1^WT^ and AP1^tet^ ChIP-seq bound genomic regions. The dashed horizontal line represents a *p*-value of 0.05. The vertical lines represent the distance ± 50 bp for visualisation

The lack of complementation of the *ap1* mutant phenotype by the tet-deficient AP1 proteins could either result from reduced DNA-binding capacity in an in vivo context, or from impaired abilities to regulate target gene activities. We therefore determined AP1-bound genomic regions at genome-wide scale by chromatin immunoprecipitation followed by deep sequencing (ChIP-seq) (Additional file 3: Table S2). Global levels of enrichment and number of significant AP1-bound genomic regions were reduced in all ChIP-seq experiments of tet-deficient AP1 mutants (Fig. 2a; Additional file 1: Fig. S5a). This could not be explained by general differences in protein levels of wildtype vs. mutant proteins (Additional file 1: Fig. S1a). To confirm that mutating the tetramerisation interface does not affect the binding efficiency of AP1 to naked DNA, we furthermore calculated the dissociation constant (Kd) for probes derived from AP1 targets (Additional file 2: Fig. S2a). We observed that AP1^tet^ and AP1^WT^ had similar DNA-binding levels and AP1^tet^ moreover demonstrated a reduced Kd for the *AP1* promoter and *AG* intron probes (Additional file 2: Fig. S2b).

Direct AP1 target genes that control floral organ specification, such as *SEP3* and *AP3,* are among the targets that lost significant AP1 binding in the tet-deficient mutants (Fig. 2b). The data indicate that tetramerisation is important for effective DNA-binding of AP1 in vivo, possibly by stabilising DNA contacts via cooperative binding to two cognate DNA-binding sites.

Previous EMSA analyses showed that MADS protein tetramers bind to DNA sites with two CArG boxes at a preferred distance, potentially resulting in DNA loop formation [13]. SELEX-seq was used to identify DNA sequences bound by AP1 homodimers in vitro*,* and the obtained kmer data were mapped to the genome to predict AP1 binding sites (Additional file 4: Table S3). AP1^WT^ ChIP-seq peaks displayed slightly higher frequencies of SELEX-predicted AP1 binding sites than AP1^tet^ ChIP-seq peak regions (Additional file 1: Fig. S5b). More strikingly, AP1^WT^-bound or AP1^WT^-GFP bound genomic regions showed a preference for specific spacing of ~ 50 bp expected for DNA-binding of tetrameric complexes, while this pattern was lost in the AP1^tet^-bound genomic regions (Fig. 2c; Additional file 1: Fig. S5c). Together, these results show that tetramerisation facilitates effective DNA-binding of AP1 in an in vivo context.

### Loss of tetramerisation affects AP1 DNA-binding to closed chromatin

Floral meristem specification, patterning, and growth require coordinated activities of TFs that together regulate timing and spatial specificity of target gene activation [14]. Indirect effects of TF perturbation, such as compensatory feedback loops, can thus impede the analysis of biochemical functions of individual TFs in this network. We therefore set up a seedling-based synthetic system (Additional file 1: Fig. S6) utilising the dexamethasone (DEX)-inducible LhGR transcription factor [15] to study the impact of tetramerisation on AP1 DNA-binding and target gene activation. We analysed phenotypes of seedlings upon DEX induction and performed ATAC-seq (Additional file 6: Table S5), ChIP-seq (Additional file 3: Table S2, Additional file 7: Table S6), and mRNA-seq (Additional file 8: Table S7) time-series experiments (Additional file 1: Fig. S7).

**Fig. 3:**
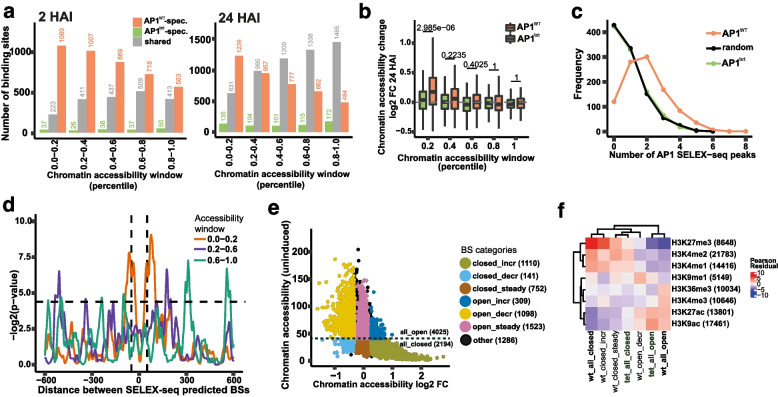
Synthetic activation of AP1 in seedlings shows that tetramerisation facilitates AP1 pioneer activities in vivo. **a** Number of AP1^WT^-specific, AP^tet^-specific, and shared bound genomic regions associated with different levels of chromatin accessibility in the uninduced mock sample at 2 h. **b** Changes in chromatin accessibility at 24 HAI (DEX/MOCK) for top 200 AP1^WT^ and AP1^tet^-specifically bound regions in seedlings with different levels of chromatin accessibility at 2 h uninduced (mock) sample, defined as percentile windows. *P*-values were obtained using the unpaired two-sided Mann–Whitney test. **c** Number of AP1 SELEX-seq peaks overlapping with the top 1,000 AP1^WT^-specific BSs, top 1,000 AP1^tet^ specific BSs, as well as with a random set of 1,000 genomic regions. **d** AP1 SELEX-seq peak enrichment (-log2 of *p*-value) at specific distances within the top 500 AP1^WT^-bound regions with different chromatin accessibility windows (red line represents closed chromatin, blue line represents open chromatin, green line represents intermediate state of chromatin accessibility) at 2 HAI. The dashed horizontal line represents a *p*-value of 0.05. The vertical lines represent the distance ± 50 bp for visualisation. **e** Classification of AP1^WT^-bound genomic regions at 2 HAI into 9 binding site (BS) categories (number of AP1 BSs shown in parentheses) according to chromatin accessibility levels at the earliest time point (2 h uninduced) and chromatin accessibility dynamics (log2 FC from 2 h uninduced to 48 HAI) as determined by a ChIP-seq and ATAC-seq time series in seedlings. Data points represent AP1.^WT^ binding sites and are coloured based on the BS category. **f** Heatmap showing the enrichment (red) or depletion (blue) measured as Pearson residuals of the overlap between particular histone modifications with AP1-bound genomic regions belonging to selected BS categories shown in e. *P*-value of Chi-square statistic = 3.17 × 10^−36

We asked how DNA-binding dynamics were affected by loss of tetramerisation upon AP1 induction in seedlings. AP1^WT^ ChIP-seq data analysis revealed an increase from ~ 6,200 significant AP1-bound genomic regions at 2 h after induction (HAI), to ~ 9,700 AP1-bound regions at 24 HAI (Fig. S9a). In contrast, ~ 2,200 genomic regions were bound at 2 HAI by AP1^tet^, with a steeper increase to ~ 6,200 regions at 24 HAI. At 2 HAI, ~ 68% of AP1 binding sites were wt-specific, while at 24 HAI, this fraction decreased to ~ 42%. AP1^tet^ is thus essentially able to access a subset of the AP1^WT^ binding sites at an early timepoint after induction. While global differences in occupancy levels could be related to reduced stability of the AP1^tet^ protein, we speculated that specific differences in AP1 DNA-binding activity result from reduced capacity of AP1^tet^ in binding to DNA in less accessible chromatin regions. Indeed, AP1^tet^ showed a reduced preponderance to bind to genomic regions that had lower read coverage in ATAC-seq compared to AP1^WT^ at 2 HAI (Fig. 3a). A similar trend was observed at 24 HAI, although the overall differences were less pronounced. In summary, tetramerisation of AP1 facilitates binding to closed chromatin, which is essential for its role as a master transcription factor promoting floral cell fate.

### AP1 tetrameric complexes function as pioneer transcription factors

In order to study AP1 binding effects on chromatin, we further analysed chromatin status changes after AP1 induction. We found that AP1^WT^ binding to closed chromatin resulted in a significantly stronger increase in chromatin accessibility at 24 HAI than AP1^tet^ binding (Fig. 3b, Additional file 1: Figure S8 a, b). AP1^WT^-bound genomic regions showed enrichment of SELEX-seq predicted AP1 binding sites compared to AP1^tet^, indicating that AP1^WT^ is able to access its DNA-binding sites more effectively because of cooperative binding to multiple DNA elements (Fig. 3c).

A role for higher-order complex formation in mediating the opening of closed chromatin was further corroborated by the preferred spacing of SELEX-seq peaks (as a proxy for MADS-domain TF binding sites) being more pronounced in AP1-bound genomic regions located in regions with low ATAC-signals (Fig. 3d). We next classified regions bound by AP1^WT^ at 2 HAI in seedlings according to their ability to bind closed chromatin and to alter chromatin accessibility using an extended time-series up to 48 HAI (Fig. 3e, Additional file 7: Table S6). 1,110 out of 2,194 ‘closed’ chromatin regions bound by AP1 were found to have a significant increase in chromatin accessibility (FC > 1.2; *p*-value < 0.1) from 2 h uninduced to 48 HAI. Interestingly, those closed AP1-bound regions that show an increase in accessibility and are occupied already in the early FM also show a preponderance for increase in accessibility during early flower development (Additional file 1: Fig. S9g).

Analysing the histone modification status in closed vs. open chromatin regions bound by AP1, as well as in the more specific categories, revealed AP1^WT^-bound genomic regions in closed chromatin are overrepresented in regions bound by repressive histone marks, especially histone-3-lysine-27 trimethylation (H3K27me3) (Fig. 3f). A second mark that was associated with these regions was H3K4me2, which was previously determined as a repressive mark in plants [16]. In contrast, AP1^tet^ binding sites associated with more closed chromatin did not show an enrichment in H3K27me3, suggesting that loss of tetramerisation impairs access to H3K27me3-compacted chromatin. Fewer differences were observed between AP1^WT^ and AP1^tet^ binding sites residing in open chromatin: both were enriched in H3K9ac and H3K27ac, while H3K4me3 and H3K36me3 were less enriched in AP1^tet^-bound genomic regions. We next asked whether potential direct AP1 target genes correspond to specific types of gene functions depending on whether they reside in open vs. closed chromatin based on the ATAC-seq data. Genes with AP1 DNA-binding sites that are in closed chromatin are enriched for gene ontology (GO) categories related to ‘development’, while potential AP1 targets in ‘open chromatin’ are enriched in GO categories related to chemical stimuli (Additional file 1: Fig. S9e). Together, the data point towards AP1 tetramerisation facilitating DNA-binding to closed chromatin regions, such as Polycomb repressive regions and promotes chromatin opening thereby controlling the activity of development-related genes.

In line with a role for AP1^WT^ to programme gene expression towards a floral fate, phenotypic analysis of LhGR/pOP6::AP1WT seedlings treated with 10 mM DEX for 10 days in soil resulted in reduced leaf size, upward ‘curling’ (as reported for constitutive AP1 overexpression, [16]), and a partial conversion of epidermal cell identities in the first emerging leaves towards sepal identity, such as the formation of abaxial trichomes (Additional file 1: Fig. S9h).

Time-series transcriptome analyses (Additional file 7: Table S6) in seedlings upon AP1^WT^ induction revealed only a relatively small number of differentially expressed (DE) genes until 24 HAI. In particular, 214, 184, and 423 genes were differentially expressed between DEX and MOCK (abs(log2FC) > 1 and FDR < 0.05) treated samples for the time-points 2, 8, and 24 HAI, respectively. 80% (2 HAI), 63% (8 HAI), and 47% (24 HAI) of these genes were activated (log2FC > 1 and FDR < 0.05). Interestingly, ~ 58% of all DE genes, and ~ 61% of the activated genes had at least one AP1 bound-genomic region in their vicinity (3 kb upstream to 1 kb downstream of the genes). This fraction of directly regulated genes by AP1 is exceptionally high, and shows the feasibility of this synthetic system to identify genes that directly respond to AP1 binding. In more general terms, the results indicate that AP1 is able to regulate a small subset of its potential direct target genes in this ectopic context, but may need regulatory interplay with flower-specific interaction partner proteins such as SEP proteins to activate other direct targets. Combinatorial binding with other (non-MADS) TFs to the same promoters in a ‘floral context’ may further be required for regulation of those genes. For comparison, 250 genes with AP1 binding sites are significantly differentially expressed within 12 h after AP1 induction in the transition from IM to FM identity (35S::AP1-GR *ap1 cal*), out of 1366 genes (18%) that significantly respond to AP1 induction at this timepoint [17]. There are only 38 genes in common between the seedling datasets and the FM datasets, such as *SEP3* and *RGA-like2* (*RGL2*). Since we observed differences in trichome patterning in AP1^WT^ vs. AP1^tet^ seedlings (Additional file 1: Fig. S9h), we identified candidate genes controlling the modification of epidermal cell identities and trichome patterning in developing leaves upon AP1^WT^ induction in seedlings. Based on differentially expressed direct AP1 target genes, the formation of abaxial trichomes can be explained by the direct repression of *TEMPRANILLO 2* (*TEM2*), a known repressor of trichome formation on the abaxial epidermis of sepals, along with the upregulation of *GIBBERELLIN 2 OXIDASE 2* (*GA2OX2*) by AP1 (Additional file 1: Fig. S9 b, c). *GA2OX2* is known to accelerate the formation of abaxial trichomes [18], and is usually repressed by TEM TFs [19]. *GA2OX2* and *TEM2*, along with *TEM1*, are also bound by AP1 in inflorescences (Additional file 1: Fig. S9d; Additional file 3: Table S2). In contrast, the number of DE genes upon AP1^tet^ induction was very low (28 genes at abs(FC) > 2 and FDR < 0.05 for 24 HAI compared to 423 genes for AP1^WT^ at the same timepoint; (Additional file 7: Table S6)). Together, the results indicate that inducible overexpression of AP1^WT^ confers sepal epidermal characteristics to early arising leaves by regulation of genes involved in trichome patterning.

In summary, these data point towards the requirement of higher-order complex formation in providing access of AP1 to closed chromatin, such as Polycomb repressive chromatin, and in chromatin opening, characteristics that qualify AP1 as a pioneer TF. Candidate partners for AP1 heterotetramerisation in seedlings are SOC1 and SVP [12]. Indeed, AP1-bound genomic regions in seedlings showed strong overlap with SOC1-bound genomic regions based on ChIP-seq data (Additional file 1: Fig. S9f). Combining the results from inflorescences and seedlings further highlights the importance of AP1 tetramers to the priming of developmentally regulated genes in the floral meristem.

### Closed chromatin regions are bound and become accessible upon AP1 binding in vivo

**Fig. 4:**
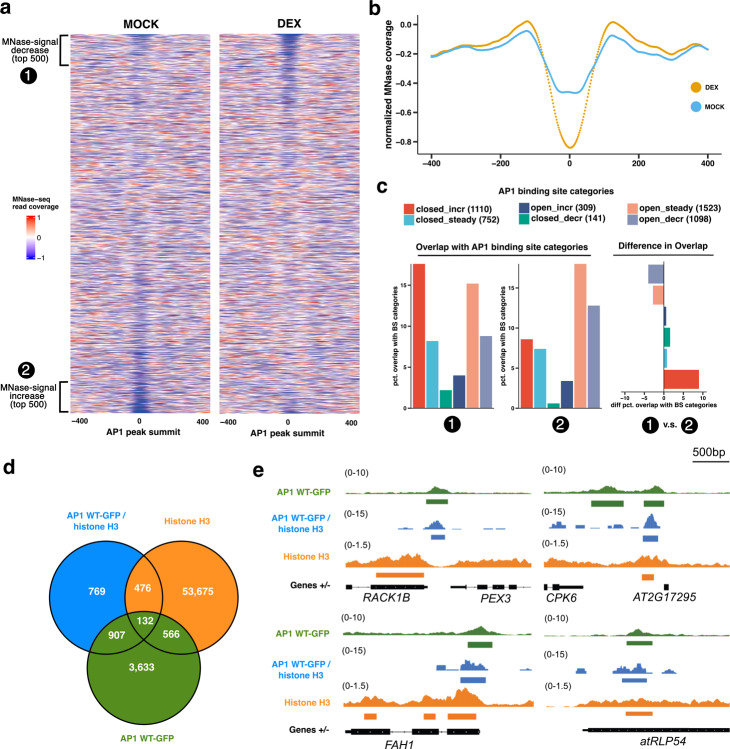
In planta AP1 activity demonstrates binding of histone H3 occupied chromatin and opening of AP1 bound chromatin. **a**. Heatmaps showing normalised MNase-seq signal of MOCK and DEX-treated 10-day old seedlings over SELEX-seq corrected AP1 BS’s with larger values indicating more MNase-seq signal (more nucleosome associated). Regions were sorted based on the ratio of MOCK/DEX MNase-seq signal at the AP1 peak summit (region +/- 40 bp around peak summit) with regions at the top having a larger MOCK/DEX ratio. Regions were divided into categories 1 and 2 corresponding to the top and bottom 500 AP1 BS’s in this ordering scheme, respectively. The top 500 AP1 binding sites are representing regions of MNase-seq (nucleosome) depletion in DEX. **b**. MNase-seq signal pile up plot of category 1, showing a depletion of MNase-seq signal in MOCK compared to DEX (AP1 present) treated plants. **c**. For regions of both categories 1 and 2, the percentage overlap with AP1 binding site categories was calculated (left and middle plot) showing distinctive overlap with previously in Figure 3e ATAC-seq based AP1 pioneer regions (“closed_incr”). To emphasise differences of AP1 binding site category distribution, the % difference shown in the left two plots was calculated for all AP1 binding site categories (right plot). **d**. Overlap of AP1^WT^ binding sites, AP1^WT^/histone H3 binding sites, and histone H3 localisation patterns in inflorescences. **e**. Genome browser tracks showing AP1^WT^-specific, AP1^WT^/histone H3-binding, and histone H3 chromatin co-occupancy. Selected regions shown were chosen from 132 overlapping regions in **d**

Binding to chromatin is a general feature of all TFs, though not all TFs can directly access their DNA-binding sites in a nucleosomal configuration [20]. To further strengthen our claim that, once bound to compacted chromatin, AP1 is involved in chromatin opening, we corroborated our ATAC-seq data with MNase-seq, employing the DEX-inducible AP1 seedling system (24 HAI). Here, we took the total number of AP1 binding sites from our AP1^WT^ 24HAI seedling dataset (corrected with SELEX-seq data) and compared the differences in signal intensity between MOCK and DEX (Fig. 4a). A depletion of MNase-seq signal at the top of the heat map in the DEX-treated plants points to a higher incidence of chromatin opening not observed in the MOCK plants (Fig. 4a). To investigate whether the signal disparity between MOCK and DEX at the top regions of the MNase-seq read coverage heat maps corresponds to AP1-mediated chromatin opening, we categorised the top and bottom 500 BS’s of the MNase-seq read coverage heat maps and plotted the mean MNase-seq read coverage between both samples. A reduction in DEX signal can be observed in the top 500 category (Fig. 4b, (Additional file 1: Fig. S10b). This points to the top 500 category corresponding to a subset of a global AP1 BS’s that undergo chromatin opening upon AP1 binding, in line with AP1’s ability to impart pioneering activity at specific loci. Moreover, we looked at the effect on chromatin opening based on our previously defined categories of AP1-bound chromatin states, taking again the top and bottom 500 categories from the MNase-seq read coverage heat maps. In concordance with our ATAC-seq results, the “closed_increasing” category specifically displayed the most striking disparity between the top and bottom 500 categories, indicating further that AP1 binding is needed to trigger accessibility changes in the chromatin (Fig. 4c).

In order to more comprehensively discriminate to what extent AP1 binds compacted chromatin and exhibits pioneering activity in native inflorescence tissues, we performed sequential αAP1 αH3 ChIP-seq (seq-ChIP-seq) in inflorescences. We defined a set of 132 high-confidence AP1 pioneer regions by defining genomic regions that overlap between the AP1^WT^-GFP ChIP-seq, the seq ChIP-seq, and a previously published histone H3 ChIP-seq dataset from the same tissue types (Fig. 4d) [21]. The genomic regions shown in Fig. 4e visualise the concurrent AP1 binding and histone H3 enrichment, indicating further that AP1 binds compacted chromatin in vivo. Together, these corroborating in vivo data additionally point to AP1’s important role during chromatin state changes in the floral meristematic stages of early reproductive development.

### AP1 binds nucleosomes in vitro

**Fig. 5:**
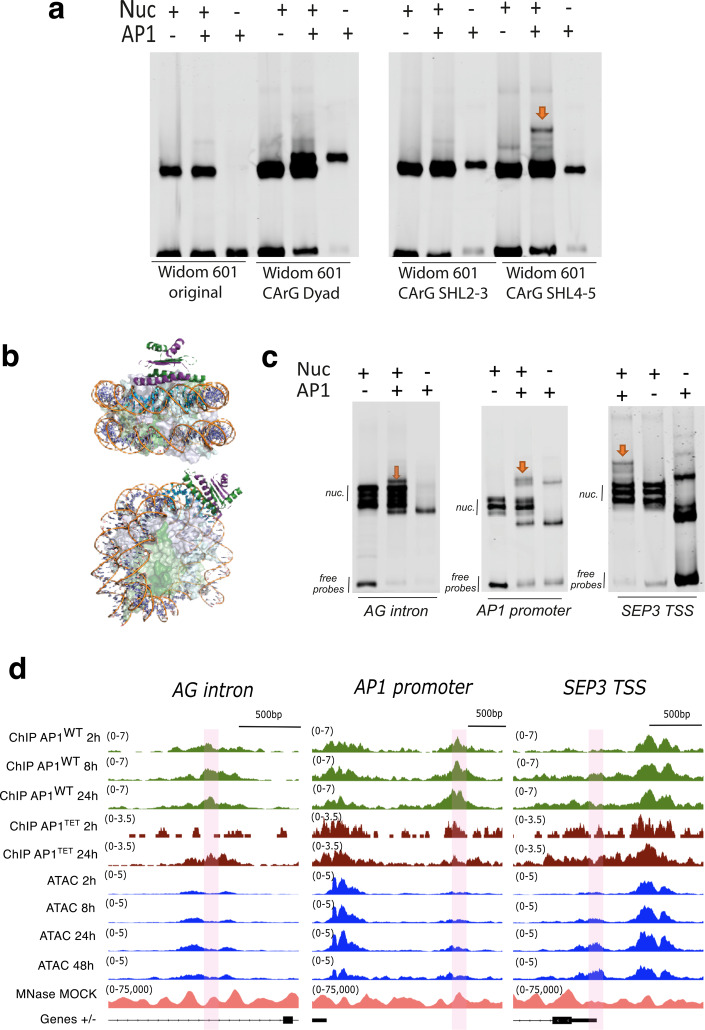
AP1 binding to CArG boxes in a nucleosomal context. **a** EMSA showing AP1 binding to Widom 601 nucleosomes with a CArG box inserted at position superhelix location 4–5 (SHL4), but not at SHL2-3, Dyad (SHL0) or the original Widom 601 nucleosome without a CArG box. **b** Structure modelling demonstrating the AP1 binding onto a nucleosome reconstituted with the Widom 601 sequence when the CArG box is inserted at SHL4-5. **c**. EMSA showing the binding of AP1 to nucleosomes reconstituted with the sequences from the AG intron, the AP1 promoter, and the SEP3 transcription starting site (TSS). **d.** Genome browser tracks of AP1^WT^ and AP1^tet^ seedling time-series ChIP-seq data (green and burgundy respectively), time-series seedling ATAC-seq data (blue), and seedling MNase-seq MOCK data at native DNA sequences chosen for nucleosome reconstitution and nucleosomal DNA binding assay. Genome browser tracks showing AP1^WT^-specific, AP1^WT^/histone H3-binding, and histone H3 chromatin co-occupancy. Selected regions shown were chosen from 132 overlapping regions in d

To study AP1 pioneer activity in vitro*,* we performed nucleosomal DNA binding assays. We introduced CArG box sequences into different positions with increasing distance to the dyad axis of the Widom 601 nucleosome positioning sequence [22]. Three insertion positions were tested: dyad (superhelix location [SHL] 0–1), SHL2-3 and SHL4-5. We observed that AP1 could bind to the CArG box when inserted into the superhelix location 4–5 (SHL4), indicating that a preferred binding conformation is required for AP1 nucleosomal binding in vitro (Fig. 5a, Additional File 1: Fig. S11 a, b). Next, using the crystal structure of the nucleosome core particle with the Widom 601 DNA sequence (PDB 3LZ0) as a model, the DNA conformation corresponding to the differentially positioned CArG boxes was examined. Structural overlays of the nucleosomal DNA with the DNA-bound MEF2A structure (PDB 1EGW), a structural homolog of AP1, revealed a preferential orientation for the CArG-box for the SHL4-5 site (Fig. 5b; Additional file 1: Fig. S12a). As shown in Fig. 5b, the CArG box is positioned with the major and minor groove bases oriented in good agreement with the crystal structure of DNA-bound MEF2A. This confirms that AP1 can bind to target sites localised in nucleosomes. We also tested native Arabidopsis promoter sequences harbouring AP1 in vivo binding sites in regions where the DNA accessibility increases after AP1 induction in seedlings with MNase-seq peaks observed at those loci in MOCK AP1 seedling plants (Fig. 5d). We observed binding of AP1 to nucleosomes reconstituted using sequences from the *AG* intron, *AP1* promoter, and the *SEP3* transcriptional start site (TSS) (Fig. 5c) containing one or more CArG-box like elements (Additional file 1: Fig. S12b). Titration experiments revealed the specificity of the shifted band corresponding to the AP1/nucleosome complex (Additional file 1: Fig. S12c). Taken together, these data support the claim that AP1 can effectively bind nucleosomes in vitro, shedding further light on the additional pioneering characteristics of this important floral meristem identity factor.

## Discussion

We provide evidence that AP1 tetramerisation stabilises general DNA-binding and facilitates pioneer factor activity *in planta*. In particular, tetramerisation enhances binding to, and opening of, compacted chromatin. The globally lower occupancy levels of tet-deficient AP1 mutant proteins, related to a lower DNA affinity of dimers than of tetrameric complexes, can affect the kinetics of target gene activation, resulting in lack of mutant complementation. While we did observe DNA-binding of AP1 homodimers to different nucleosome-bound genomic regions in vitro, DNA-binding of the AP1^tet^ mutant protein to closed chromatin in vivo was reduced. This suggests a model according to which simultaneous DNA-binding to two CArG boxes can provide or stabilise access of MADS TF tetramers to the DNA and enable effective engagement in closed chromatin. This two-site binding may mediate nucleosome displacement in vivo, supporting a previous theoretical calculation based on in vitro MADS TF-DNA dissociation constants [6]. Furthermore, AP1 is not only able to form heterotetramers with homeotic TFs, but also with regulators of floral transition that provide a repressive activity to the complex, especially SOC1 and SVP. The prevalence of repressive AP1-containing complexes in seedlings, as indicated by *SOC1* and *SVP* expression, can be one reason to explain the relatively low number of genes bound by AP1^WT^ that are activated in our seedling transcriptomics time course experiments. SVP was previously shown to interact with the Polycomb Group protein TERMINAL FLOWER 2/LIKE HETEROCHROMATIN 1 (TFL2/LHP1) [23] that confers a repressive state to genomic loci marked by H3K27me3 [24]. On the other hand, SOC1 was found to interact with the histone deacetylase complex component SIN3 ASSOCIATED POLYPEPTIDE 18 (SAP18) [23]. Together, this suggests that SOC1 and SVP confer repressive potential to AP1 via interactions with epigenetic regulators, thus antagonising the pioneer activity required for initiation of gene activities. Future research should address the competitive activities of activating and repressing AP1 complexes, e.g. by analysing chromatin and gene expression changes upon simultaneous induction of AP1 and specific partners in the synthetic seedling system, and by more targeted assays that allow the variation of concentrations of repressive and activating tetramers. Additionally, DNA-bending and tetramer-specific loop formation can be expected to alter promoter 3D architecture, thereby shifting the relative positions of cis-regulatory regions and of RNA polymerase II complexes.

Our and others’ previous work has shown that a variety of MADS domain heterotetramers can be formed. Tetramerisation may affect target gene specificity, because individual dimers in a complex can differ in DNA-binding specificity and different tetramers may prefer distinct spacing of CArG boxes for optimal binding [6]. Here we show that TF tetramerisation allows effective DNA-binding in vivo and assists in chromatin binding and opening. The question remains whether the pioneer activity is specific to AP1 (and possibly evolutionarily closely related factors such as the SEP proteins), or whether it is a general feature of plant MIKC-type MADS domain proteins. A conserved role for tetramerisation in providing access to closed chromatin could explain why these factors have acquired diverse functions in developmental phase transitions and organ specification in plants. Intriguingly, the evolution of tetramerisation near the origin of land plants may have provided an increased capacity of these proteins to bind DNA in an in vivo chromatin context, thus contributing more complex developmental decision processes associated with the evolution of the land plant life cycle.

## Conclusions

In this study, we identified AP1 as a pioneer transcription factor in addition to the mechanistic basis underlying its pioneer activity. We show that AP1 has the ability to bind compacted chromatin and that tetramerisation enhances the binding to and subsequent opening of compacted chromatin. The important contribution of tetramerisation to AP1 pioneer activity was further supported through the use of tetramerisation deficient AP1 mutant proteins, which exhibited a reduction in genome-wide occupancy levels compared to the AP1 WT protein. The inability of AP1 to tetramerise can affect the local 3D chromatin architecture of the target locus, thereby preventing effective recruitment of further necessary players of the transcriptional apparatus, as well as the kinetics of target gene activation. This ultimately manifests in a lack of mutant complementation. Although a degree of DNA binding of AP1 homodimers to different nucleosome-bound genomic regions in vitro was observed, DNA-binding of the AP1^tet^ mutant protein to closed chromatin in vivo was reduced. These findings thus suggest a model where local two-site binding, contingent on tetramerisation, confers pioneering activity in vivo whereby the tetramer provides stability in the binding to closed chromatin and effective engagement to open it. These mechanistic insights into higher-order complex mediated chromatin state changes and ensuing gene activity are widely relevant and will further drive our understating of tightly controlled developmental programmes underlying cell fate decisions in eukaryotes.

## Methods

### Cloning and plant transformation

To introduce the GFP-fused AP1^WT^ and AP1^tet^-deficient genomic mutant constructs into the *ap1*-*11* background plant, native AP1 genomic sequences with its promoter region (3,207 bp upstream of ATG) were amplified from *Col*−0 or *ap1*^*tet*^ (*ap1* CRISPR mutant without exon 5 and 6) genomic DNA and cloned into the pCR8 vector (Thermo Fisher) by TA cloning. The AP1^dm^ construct was generated by PCR-directed mutagenesis using the AP1^WT^ promoter + genomic locus cloned into pCR8 as a template. AP1^WT^, AP1^tet^ and AP1^dm^ sequences (data S7) were transferred into pMDC107 destination vector harboring a GFP tag using LR reaction and ultimately transformed into *ap1-11* with the floral dip method via *A. tumefaciens* cells as described [25].

To induce the expression of *AP1* in seedlings, an activator construct and a reporter construct of the pOp/LhGR induction system [15], were transformed into *Col*−0 plants. The *UBQ10* promoter was inserted in front of the *LhGR* coding sequence by gateway LR reaction to generate the activator construct. In addition, The *AP1* coding sequence was cloned to the reporter construct carrying the pOp6 promoter by restriction enzyme digestion and ligation.

The C domain-deleted AP1 and SEP3 coding sequence (data S7) was cloned into pGEX-6P1 containing a GST tag for protein expression in *E. coli*.

### Plant materials and growth conditions

Sterilised seeds were either sown on agar medium or soil supplemented with vermiculite and stratified at 4 °C in the dark for 2–3 days. Seeds were then transferred to 22℃ at 60% humidity and grew subjected to long-day conditions (16 h light, 8 h dark) until ready for tissue collection.

AP1 inducible seedlings were grown on ½ MS medium containing 1% sucrose and were induced by spraying dex solution (25 μl 20 mM dexamethasone in DMSO and 8 μl Silwet in 50 ml water). The mock control seedlings were sprayed with mock solution (25 μl DMSO and 8 μl Silwet in 50 ml water). The aerial parts of the plants were then collected at different time points after induction. For the leaf cell phenotyping, 10-day-old seedlings grown in soil were sprayed with DEX solution or MOCK solution for control once a day for 10 days.

### Phenotyping

Inflorescences and mature flowers of all plants employed in this study were phenotyped by mounting in charcoal supplemented agar plates (1.5 g phytoagar, ~ 1 g activated charcoal, and 100 ml H_2_O) and imaged with a Leica DMV6 camera. Z-stacks were taken for each specimen and maximum projected to generate high-resolution processed images. Plants at a uniform developmental stage were chosen for imaging.

To perform phenotyping of leaf cells after AP1 induction, rosette leaves were removed from plants and fixed by methanol and ethanol according to a published protocol [26]. Fixed leaves were critical-point dried and mounted to a stub for gold sputtering. Phenotyping was done by scanning electron microscopy with a working distance of 10 mm and the Extra High Tension of 20 kV.

### Confocal imaging

Arabidopsis inflorescences were dissected under a stereo microscope, immobilized in 0.15% agar inside a microscopic chamber and covered with a cover slide. AP1-GFP tagged protein localisation was observed using ZEISS LSM 800 upright confocal microscope with C-Apochromat 40x/1.2 W Korr objective. The GFP was excited with the 488 nm laser and the emission signal was detected between 410 and 525 nm while the chlorophyll autofluorescence signal was detected between 650 and 700 nm. Images of individual optical slices (Z-stacks) were smoothed using “Lowpass” method (settings: Kernel Size x = 3, y = 3, z = 1) and merged by “Orthogonal Projection” method (settings: Maximum, Frontal XY plane) of the ZEISS Zen Blue (version 2.1) software.

For early stage flower imaging, inflorescences were incubated for 2 min in 1 mg/ml propidium iodide (PI) solution to stain cell walls of undamaged cells. AP1-GFP tagged protein localisation was observed with C-Apochromat 63x/1.2 W Korr UV VIS IR objective. The PI was excited with the 561 nm laser and the emission signal was detected between 450 and 700 nm. Laser intensities were adjusted for the best signal and individual optical plane images (Z-stacks) were recorded using super resolution Airyscan mode. The raw scan data was post-processed with the “Airyscan processing” method and the image display was adjusted to accommodate a lower dynamic range of pixel intensities.

### Electrophoretic mobility shift assay

Electrophoretic mobility shift assays (EMSAs) were performed as previously described [5]. Briefly, the oligonucleotide sequences, cloned into pGEM-T vector (Promega), were amplified using vector-specific, 5’-DY682-labeled primers. The coding sequences of MADS-domain proteins in the pSPUTK (Stratagene) expression vector were either available in house or amplified from a pool of cDNA and cloned into pSPUTK. Proteins were synthesised using the TnT SP6 High-Yield Wheat Germ Protein Expression System (Promega) according to the manufacturer’s instructions in a total volume of 10 μl. To study formation of protein-DNA complexes, protein components were synthesised together in a single tube where equimolar plasmid concentrations were used. The binding reaction mixture contained 1.2 mM EDTA (pH 8.0), 0.25 mg/ml BSA, 7.2 mM HEPES (pH 7.3), 0.7 mM DTT, 60 μg/ml salmon sperm DNA, 1.3 mM spermidine, 2.5% CHAPS, 8% glycerol, 3.3 nmol/ml oligonucleotide fragments, and 2 μl of in vitro-synthesised proteins. The binding mixture was incubated on ice for 30–45 min and loaded on a native 5% polyacrylamide gel (1 × TBE). The gel was run at 75 V for 90 min at room temperature, followed by direct signal detection on the Odyssey DLx Imager (Li-Cor).

### Protein immunoprecipitation

Immunoprecipitation of GFP-tagged protein complexes followed by liquid chromatography-tandem mass spectrometry (IP-MS) and subsequent data post-processing was performed largely as described [27]. Plants were grown under long day conditions (16 h of light/8 h of dark at 21 °C) on soil. Approximately 1.0 g of primary and secondary inflorescences with unopened flowers were collected, immediately frozen in liquid nitrogen, and ground using a pestle and mortar. The frozen tissue powder was resuspended in 20 ml M1 buffer (10 mM sodium phosphate buffer pH 7.0, 0.1 M NaCl, 1 M 2-methyl 2.4-pentanediol, 10 mM β-mercaptoethanol, 1 × cOmplete protease inhibitor cocktail), filtered through a 55-µm nylon mesh and centrifuged at 1,000 g for 20 min at 4 °C. The pellet was resuspended in 5 ml M2 buffer (M1 buffer with 10 mM MgCl_2_, 0.5% Triton X-100) and centrifuged at 1,000 g for 10 min at 4 °C. The wash with M2 buffer was done five times in total. The pellet was resuspended in 5 ml M3 buffer (M1 buffer without 2-methyl 2,4-pentanediol), centrifuged at 1,000 g for 10 min at 4 °C and further resuspended in 1 ml Lysis Buffer (μMACS GFP Isolation Kit; Miltenyi Biotec) supplemented with 1 × cOmplete protease inhibitor cocktail and 0.125 U/µl Benzonase nuclease, purity > 99% (Novagen). The lysate was sonicated using a probe sonicator (Covaris S220) with the following settings: Peak Power = 140.0; Cycles/Burst = 200; Duty Factor = 5.0; Duration = 360 s; temp = 6 °C. The protein lysate was cleared 2 times by centrifugation at 20,000 g for 10 min at 4 °C. For the input sample preparation and for the total protein amount estimation, 35 µl of the supernatant (soluble protein extract) was set aside. The rest of the soluble protein extract was mixed with 50 µl anti-GFP beads (μMACS GFP Isolation Kit; Miltenyi Biotec) and incubated on a tube rotator at 10 rpm for 1 h at 4 °C. The µ-Column (Miltenyi Biotec) was placed in the μMACS Separator (Miltenyi Biotec) and equilibrated with 200 µl cold Lysis Buffer. The mix was applied to the magnetic column and the beads were washed 5 times with 200 µl Lysis Buffer and 2 times with 200 µl Wash Buffer 2 (μMACS GFP Isolation Kit; Miltenyi Biotec) followed by incubation with 20 µl of 8 M urea for 5 min at room temperature (RT). Immunoprecipitated proteins (IP sample) were eluted from the column with 50 µl 8 M urea. Proteins were reduced with 5 mM DTT for 30 min at 56 °C with 700 rpm in the thermomixer, alkylated with 10 mM iodoacetamide for 30 min at RT in the dark and diluted to the final urea concentration at 1.6 M with 50 mM ammonium bicarbonate (ABC buffer). Proteins were digested with 0.5 µg of trypsin (Trypsin Gold, Mass Spectrometry Grade, Promega) at 37 °C overnight (O/N). Peptide sample was acidified with 1% formic acid (FA) and desalted using Oasis HLB 96-well µElution Plate (Waters) using two 300 µl washes with 1% FA and eluted 2 times with 50 µl 50% acetonitrile (ACN)/0.5% FA solution. Eluted peptide solution was dried down using a SpeedVac for 1.5 h at 60 °C with 5.1 mTor. Dried peptides were stored at −20 °C.

Total protein amount in the soluble protein extract was estimated using Micro BCA Protein Assay Kit (Thermo Fisher Scientific) following manufacturer's instructions. Input sample was processed with the modified filter-aided sample preparation (FASP) method [28]. 200 µl 8 M urea in 50 mM ammonium bicarbonate (UA buffer) was added to 25 µg of protein (at approximately 1 µg/µl). Proteins were reduced with 10 mM DTT for 30 min at 56 °C with 700 rpm in the thermomixer and alkylated with 20 mM iodoacetamide for 30 min at RT in the dark. Protein solution was transferred to the Amicon Ultra-0.5 Centrifugal Filter 30 kDa MWCO (Millipore), centrifuged at 14,000 g for 10 min at RT, then, washed 3 times with 200 µl UA buffer and 3 times with ABC buffer each time centrifuging in between. The filter unit was placed in a new collection tube and 100 µl ABC buffer and 0.5 µg of trypsin (Trypsin Gold, Mass Spectrometry Grade, Promega) was added and incubated in a wet chamber at 37 °C O/N. Input sample was eluted from the filter by centrifugation and two washes with 100 µl ABC buffer each time followed by centrifugation. The peptide-containing filtrate was acidified with 1% FA and desalted in the same way as the IP sample.

### Liquid chromatography-mass spectrometry

Dried peptides were resuspended in 25 µl 0.1% FA/2% ACN, vortexed and sonicated in a water-bath sonicator for 10 min at RT. Peptide concentration was estimated using NanoDrop One A205 method. Samples were analysed using the EASY-nLC 1200 high-performance liquid chromatograph (Thermo Fisher Scientific) coupled to the Q Exactive Plus mass spectrometer (Thermo Fisher Scientific). Approximately 2 µg of peptides were loaded onto the Acclaim PepMap 100 C18 (75 μm inner diameter, 15 cm long, 2 µm particle size, Thermo Fisher Scientific) analytical column with 5% (v/v) solvent B (80% ACN/0.1% FA) in solvent A (0.1% FA) and separated with a linear solvent B gradient of 5% to 40% over 70 min and 40% to 50% over 5 min followed by a washing step with 100% solvent B for 10 min and an equilibration step with 5% solvent B for 5 min at a flow rate of 300 nl/min resulting in a total 90-min run. Full MS spectra were acquired with a scan range of 300–1650 m/z, resolution of 70,000, AGC target of 3e6 and maximum IT of 50 ms. Tandem MS spectra (MS/MS) were acquired in the data-dependent acquisition (DDA) mode using a TOP15 method with a scan range of 200–2000 m/z, resolution of 17,500, AGC target of 1e5, maximum IT of 50 ms, isolation window of 2.5 m/z and normalised collision energy at 25%. Only peptide reaching intensity threshold of 3.4e4, with + 2, + 3, + 4, + 5, + 6 charge state were selected for MS/MS fragmentation. Dynamic exclusion was set to 30 s to prevented repeated selection of MS precursors.

### Proteomics data analysis

The RAW peptide sequencing data obtained from the Q Exactive Plus were processed by the MaxQuant (version 1.6.14.0) software [29] that integrates peptide identification and quantification. For IP vs INPUT comparisons all samples were processed together, while for IP vs IP comparisons only IP samples were processed by the MaxQuant. Settings of the MaxQuant were mostly kept as default with “label-free quantification (LFQ)”, “iBAQ” and “Match between runs” options on. The protein database was Arabidopsis thaliana UniProt UP000006548 without protein isoforms. After the MaxQuant analysis, the output proteinGroups.txt file was taken for further filtering and statistical analysis in the Perseus (version 1.6.15.0) software [30]. The protein LFQ intensity data was log2-transformed before analysis. Proteins identified by only modified peptides (“Only identified by site”), by reverse sequence database (“Reverse”) and protein contaminants (“Potential Contaminant”) were removed from the analysis. Proteins with LFQ intensity values in at least two biological replicates and proteins with at least two unique peptides were kept. Missing protein LFQ intensity values were replaced only in the INPUT samples by the imputation from the left arm of the LFQ intensity normal distribution (0.3 width, 1.8 down shift, separately for each column) to emulate background protein level. Relative protein amount differences between conditions were evaluated by two-sample t-tests with permutation-based FDR correction and S0 parameter set to 1.0 [31]. The data were visualised by a volcano plot where -log_10_(*p*-values) are plotted against log_2_(protein abundance ratios) with different FDR thresholds.

### Chromatin immunoprecipitation followed by sequencing (ChIP-seq)

ChIP was done largely following [32] with several modifications mentioned below. 2.5 g to 3.5 g aerial parts of seedling leaves or 0.7–1.7 g inflorescences were used as starting materials. For immunoprecipitation, 3–6 μl of AP1 native antibody or GFP antibody (ab290, Abcam) was added to the lysate, which was then incubated at 4 °C for 1 h. 50 μl Dynabeads protein A (Thermo Fisher) was added to the lysis for another 1 h incubation at 4 °C. Dynabeads with protein-DNA complexes were washed by 1 ml low salt buffer 3 times, 1 ml high salt buffer 2 times, 1 ml LiCl buffer 1 time, and in the end 1 ml TE buffer 1 time. Each wash was done by rotation (10 rpm) for 5 min in a cold room. Finally, the protein-DNA complex was eluted with 150 µl elution buffer (1% (wt/vol) SDS, 50 mM Tris–HCl pH 8, 10 mM EDTA, 50 mM DTT) on a 70 °C mixer at 1000 rpm. ChIP-seq library preparation was performed according to the manual of the ThruPlex kit (Takara). The optimal amplification cycle for the library amplification was determined by a real-time qPCR following the manual. For DNA sequencing, 100–700 bp DNA fragments were size selected by AMPure XP beads.

### ATAC-seq experiments

Around 60 aerial parts of seedlings were collected and fixed by 1% formaldehyde for nuclei isolation following a published protocol [32]. Nuclei isolation followed by FANS was essentially performed as previously described [33]. 50,000 nuclei were used for tagmentation by incubating with 2.5 µl Tn5 in a 50 µl reaction at 37 °C for 30 min with gentle shaking at 1,000 rpm. Tagmentation was stopped by adding 50 µl EDTA buffer (50 mM Tris–HCl pH 8.0, 100 mM NaCl, 0.1% SDS and 100 mM EDTA) and incubating at 55 °C for 10 min. For de-crosslinking, 1 µl Proteinase K (20 mg/ml) was added, and incubation was done at 65 °C overnight. Before PCR amplification, DNA was purified by DNA Clean & Concentrator-500 (Zymo) and eluted using 20 µl elution buffer. Following a published protocol [34], DNA was amplified by PCR. A qPCR was done with 5 μl of pre-amplified DNA to determine the amplification cycle number, and 1/3 of the linear amplification curve was referred to. DNA fragments were purified and size-selected (200–1000 bp) by AMPure XP beads and paired-end sequenced aiming at 30 M reads per sample.

### mRNA-seq experiments

Around 5 aerial parts of seedlings were homogenised by the Precellys Homogenizer (4,000 rpm for 30 s) in a 2 ml tube with 5 glass beads and 250 μl Trizol. Another 250 μl Trizol was added and mixed well with the homogenised tissue. Tissue debris was removed by centrifugation at 12,000 g for 10 min at 4 °C and then the supernatant was then transferred to a new tube for RNA isolation following the steps described [33]. RNA pellet was finally resuspended in 30 μl nuclease-free water. RNA integrity was checked by TapeStation and concentration was measured by the Qubit. Following the manufacturer’s instructions, the RNA was then used for library preparation with the TruSeq Stranded mRNA kit (Illumina) or sent to Novogene for library preparation and sequencing using the NEBNext® UltraTM Directional RNA Library Prep Kit for Illumina® (NEB, USA) following manufacturer’s protocol**.**

### MNase-seq experiments

We largely followed the published MNase-seq protocol for plants [35]. Dex treatment was done for 24 h, and then 2–3 g AP1 inducible seedlings were collected and the same amount of untreated seedlings were collected as the mock sample. Tissues were ground into powers in liquid nitrogen. Tissue powders were first resuspended in NIB (10 mM Tris–HCl, 80 mM KCl, 10 mM EDTA, 0.5 M sucrose, 1 mM spermidine and 1 mM spermine) and then filtered through a 55 μm mesh for nuclei isolation. After being washed three times with NWB (NIB containing 0.5% of freshly prepared Triton X 100) and one time with MNB (10% sucrose, 50 mM Tris–HCl pH 7.5, 4 mM MgCl2 and 1 mM CaCl2), the isolated nuclei were resuspended in MNB. Nuclei were then distributed into 200 μl equal aliquots and various amounts of MNase with a specific enzyme unit were added to each tube (4000, 2000 and 1000 U). MNase digestion was done in a 37 °C water bath for 10 min and reactions were stopped by adding 16 μl of 0.5 M EDTA. RNA was digested by adding RNase A to the reactions. DNA was them purified using an equal volume of phenol, phenol: chloroform mixture (1:1), and chloroform, respectively and then precipitated by ethanol and NaAC. The DNA pattern was then checked using TapeStation and samples containing approximately 20% nucleosome dimers and 80% mononucleosomes were selected for library preparation. Library preparation was done with the NEB Next Ultra II DNA Library Prep Kit following the instructions of the kit manual and small fragments below the size of monmucleosomes were removed by size selection.

### Sequential ChIP-seq experiments

The protocol was developed from the standard ChIP-seq protocol we described above. 1.5 g of inflorescence material was collected and nuclei were extracted in the same way as the standard ChIP-seq. The first immunoprecipitation was done with AP1 antibody from rabbit and protein A Dynabeads. The beads washing step was the same as the standard ChIP, while the first elution was done using 50 μl non-denaturing acidic elution buffer (200 mM glycine pH 2.5) by constant pipetting for 30–60 s at room temperature. The supernatant was transferred to a new tube and 5 µl neutralisation buffer (1 M Tris pH 10.4) was added to neutralise the eluate. For the second immunoprecipitation for H3, 1.5 ml IP buffer and 3 µl H3 mouse monoclonal antibody (Proteinlab, 68,345–1) were added to the eluate. After incubation of 1 h, 50 μl anti-mouse IgG beads (ThermoFisher, 11,033) were added to the tube. After the supernatant was removed, beads were once with low salt buffer and once with TE buffer. Protein-DNA complex was eluted with 150 µl elution buffer (1% (wt/vol) SDS, 50 mM Tris–HCl pH 8, 10 mM EDTA, 50 mM DTT). NaCl was added to 250 mM for overnight decross-linking. DNA was precipitated using ethanol plus sodium acetate and washed twice with 70% ethanol. The DNA pellet was dissolved in 20 μl of water and 10 μl was used for library preparation using ThruPlex kit (Takara).

### Protein expression and purification

The pGEX-6P1 protein expression construct was transformed into the *E. coli* Arctic Express strain. Bacteria were grown in LB medium supplemented with Carbenicillin on a 37 °C shaker (220 rpm) up to OD 600 = 0.6–0.8. Cultures were cooled down on ice for > 10 min before adding 0.1 mM IPTG and then were transferred to 16 °C for overnight incubation. Bacteria were pelleted and resuspended in PBS buffer before sonication on ice. Bacteria lysate was centrifuged and filtered through a 0.45 µm membrane. The filtered supernatant was incubated at 4 °C with Sepharose 4B agarose beads on a rotator for 1 h. The mixture was then loaded onto a 5 ml pre-cold purification column and beads were sedimented by gravity. Beads were then washed with 5 ml PBS five times and 5 ml cleavage buffer (50 mM Tris–HCl pH 7.5, 150 mM NaCl, 1 mM EDTA pH 8.0, 1 mM DTT) once. Beads were then resuspended in 1 ml cleavage buffer and transferred to a 1.5 ml tube. Beads were pelleted at 500 × g, 4℃, 5 min and 700 μL supernatant was removed. 20 μL PreScission protease (40 U) was added to the beads mixture for overnight cleavage of the GST tag at 4 °C. The beads mixture was loaded onto a new column, and flow-through containing proteins cleaved off the GST tag was collected. The beads were washed three times with 1 ml of PBS and all eluted fractions were collected in the Amicon Ultra-4 Centrifugal Filter Unit, 10,000 NMWL (Merck) followed by concentration by centrifugation at 5,000 xg at 4 °C until the protein concentration was around 1 mg/ml. Protein concentration was estimated by the Qubit Protein Assay (Thermo).

### Nucleosomal DNA binding assay

Assembled human histone octamers consisting of two each of histones H2A, H2B, H3 and H4 were purchased (Epicypher). Modified Widom 601 nucleosome positioning sequence with CArG-box binding motifs and native nucleosomal DNA sequence derived from *AP1* and *SEP3* promoters and *AG* second intron (data S7) were cloned into pGEM-T vector (Promega) and then labelled by DY-682 at the 5' of each DNA strand using PCR amplification. PCR products were purified by ethanol precipitation using 2.5 × volume 100% EtOH, and 1/10 volume of sodium acetate (3 M, pH 5.4) followed by incubation at −20 °C for 1 to 2 h and centrifugation at top speed for 30 min at 4 °C. DNA pellet was resuspended in 2 M NaCl. Nucleosomes were reconstituted by combining 25 pmol DNA probes and histone octamers (1:1 molar ratio) in a buffer containing 2 M NaCl, 10 mM Tris–HCl pH 7.5, 1 mM EDTA pH 8.0 and 1 mM DTT and stepwise dialysis with decreasing concentration of NaCl (1.5 M, 1 M, 0.6 M and 0.25 M) in a Slide-A-Lyzer MINI Dialysis Device, 10 K MWCO (Thermo). The final volume is around 60 μL after dialysis.

MADS-domain protein homo- and heterocomplexes were assembled by mixing 5 µg of each protein, unfolded by incubation in 6.4 M Urea, 10 mM Tris–HCl pH 7.5, 1 mM EDTA pH 8.0 and 1 mM DTT for 2 h on ice and refolded by dialysis as described above for nucleosomes. Binding of MADS-domain protein complexes to naked DNA and nucleosomes was done in binding buffer containing 10 mM Tris–HCl pH 7.5, 50 mM NaCl, 1 mM DTT, 0.25 mg/mL BSA, 2 mM MgCl2, 0.025% Nonidet P-40 substitute, 5% glycerol as described [36]. Protein concentration was measured by Qubit Protein assay kit. 5–10 μM AP1 protein was added to 1 μL of reconstituted nucleosome solution, resulting in a 10 μL reaction. Reactions were then incubated at room temperature for a defined time period, as mentioned in the main text, followed by gel electrophoresis in 5% non-denaturing polyacrylamide gel in 0.5 × TBE buffer (nucleosomal DNA binding assay) or 1 × TBE buffer (nucleosome displacement assay) at 100 V until the free DNA probes reached the bottom of the gel.

### Kd estimation

Proteins were produced in vitro using TNT SP6 High-Yield Wheat Germ Protein Expression System. Labelled DNA fragments were amplified by PCR and purified from a gel. A series of EMSA binding reactions was performed using a constant amount of protein and a changing concentration of DNA fragments. Ten serial dilutions of DNA fragments were prepared, with concentrations in the binding reaction ranging from 12.38 nM to 0.02 nM for AG intron and from 51.06 nM to 0.10 nM for AP1 promoter DNA fragments. Concentrations of “Bound” and “Free” DNA fragments were estimated from the EMSA gel band intensities. Dissociation constants (Kd) were estimated with the drc (Dose–Response Curve) R package using two separate models: a Michaelis–Menten model (Bound ~ Total) and a Scatchard plot (Bound/Free ~ Bound).

### SELEX-seq experiments

To generate input double-strand DNA (dsDNA) for the SELEX experiment, we performed a single-cycle PCR amplification using a pool of synthesised-single strand DNA (ssDNA) and complementary SELEX_PCR primers. The synthesised ssDNA pool was designed to contain 20 random nucleotides in the middle and was flanked by constant adapters of 24 nt and 22 nt at the 5’ and 3’ ends, respectively (data S7). These adaptors were compatible with the indexed adapter primers for Illumina library preparation. For protein generation, the coding sequence of AP1 was cloned into a modified pTNT vector (Promega) containing a 3 × FLAG-tag. The AP1 protein with 3XFLAG-tag was synthesised using the TNT® SP6 High-Yield Wheat Germ Protein Expression System (Promega) in a total volume of 20 µl, following the manufacturers’ instructions.

For each SELEX round, the protein-DNA binding reaction mix was prepared as studied before with a total volume of 120 µl [13], the mix contained 20 µl in vitro synthesised AP1 protein and 50 ng dsDNA. The binding reaction mix was incubated on ice for one hour, followed by immunoprecipitation using 10 µl Anti-FLAG® M2 magnetic beads (Sigma-Aldrich) in a thermomixer at 4ºC at 800 rpm for one hour. After immunoprecipitation, magnetic beads were washed five times with 150 µl binding buffer without salmon-sperm DNA and briefly rinsed with 500 µl 1 × TE buffer. Bound DNA was elucidated in 50 µl 1 × TE in a 90ºC thermomixer at full speed (1,400 rpm) for 20 min. Next, magnetic beads were immobilized by a magnet rack and the supernatant (eluate) was transferred to a new tube. To prepare for a subsequent round of SELEX, the eluate DNA was amplified by PCR using SELEX_PCR primers (data S7), and the cycle number was determined before PCR amplification using qPCR analysis. Specifically, we took out 5 µl of the PCR reaction mix and added 0.3 µl 1000 × SYBR Green Master Mix (Thermo) to conduct the qPCR, and the cycle number was chosen when the amount of DNA amplification reached or just before reaching the plateau [37]. The quality and concentration of the amplicon were checked by TapeStation (Agilent) and Qubit® Fluorometer (Thermo) respectively. For the subsequent round of SELEX, 50 ng of the amplicon was used for AP1 protein.

For high throughput sequencing, round-specific indexed adapter primers (data S7), which contain all features needed for direct sequencing, were used for library amplification by PCR. The PCR cycle number used here was also selected by qPCR before PCR amplification. All the SELEX sequencing libraries were mixed in equimolar amounts, sequencing was performed on NovaSeq 6000 (Illumina).

### SELEX-seq analysis

Two pair-end reads sequencing libraries were generated using the SELEX-seq methodology, one representing the original random pool of DNA oligos and a second one representing the DNA oligos present after 4 rounds of enrichment using AP1. As both reads of each pair-end reads represent the same 20 bp DNA sequence, any nucleotide called N in one of the reads was substituted by the corresponding nucleotide in the other read. After, when the reads of a pair-end read represent different sequences, they were filtered out. SELEX analysis was done with the R package SELEX (v1.26.0) [38], using a kmer length of 12, markov chain order of 6 and minCount of 10. This produces a table of kmer sequences and their corresponding relative DNA binding affinity to AP1. The relative DNA affinities were mapped into the *Arabidopsis* genome as in ref [39]. Namely, we mapped the 12-mer sequences to the Arabidopsis nuclear genome using the Bioconductor R package Biostrings. For each SELEX experiment, we identify regions with a high DNA binding specificity in a similar way as ChIP–seq analysis. That is, after mapping the 12-mer sequences to the nuclear genome, candidate peaks are defined as genomic regions with DNA specificity values bigger than the median specificity of all 12-mer sequences (data S4). Candidate peaks separated by less than 3 bp were merged, only regions with more than one k-mer mapped were kept. The score associated with each peak was calculated as the average DNA binding specificity of all k-mers mapped to that region for each SELEX-seq experiment.

### Primary ATAC-seq and ChIP-seq data analysis

All ChIP-seq (including sequential ChIP-seq of AP1 and histone H3) and ATAC-seq datasets generated in this project or publicly available were processed in the same way starting with raw read quality assessment, alignment to the genome and post-alignment processing.

In particular, the quality of the raw data (.fq files) was evaluated with FastQC [40] (v. 0.11.9). Then Trimmomatic [41] (v. 0.39) was used to remove adapters with default parameters. Next, reads were mapped to the *A. thaliana* genome (TAIR 10) using Bowtie2 [42] (v. 2.4.4) with default parameters. Duplicated Reads were marked using Picard tools [43] (v. 2.27.5). Subsequently, samtools [44] (v. 1.13) was used to convert aligned reads (.sam) into.bam format, sort reads by coordinates, remove unmapped reads, remove reads failing platform quality checks, remove reads with a mapping quality < 1 (a mapping quality score < 40 was used for ChIP-seq datasets in inflorescences), and remove reads marked by Picard as duplicated reads in the previous step. For further analysis, biological replicates of mapped reads were merged using “samtools merge”. MACS (v. 3.0.0b1) [45] was used to perform peak calling with the parameters -q 0.1, –mfold 1 50 and other parameters left at their default value (data S2). In the case of ChIP-seq datasets, the parameter -c was used specifying control samples. In seedlings, uninduced (mock) samples were used, while in inflorescences mock IPs on the *ap1*-*11* mutant (AP1 antibody) or pUBQ10:NLS-GFP (GFP antibody) were used. For peak calling of H3 and sequential ChIP-seq data, q was set to “0.5” to more accurately capture the peak profile of this dataset.

To visualise the coverage of aligned reads, the command “bamCoverage'' of the deeptools package [46] (v. 3.5.1) was used with the parameters bs 1, –skipNonCoveredRegions, –normalizeUsing CPM.

### Combining ChIP-seq data

ChIP-seq peak regions obtained by MACS were combined by merging regions when they overlap at least 1 bp in a set of samples of interest using functions from the GenomicRanges package (v. 1.46.1) [47] in a custom script.

In the case of ChIP-seq data in seedlings, peak regions for different genotypes (AP1^WT^; AP1^tet^) and time points (2 HAI; 8 HAI; 24 HAI), were merged to obtain a collection of potential AP1 binding sites in seedlings (data S5). In the case of ChIP-seq data in flowers, for each mutant-approach (AP1^dm^-GFP, AP1^tet^-GFP, and AP1^tet^) one set of merged regions was generated based on the respective individual peak region.

### Integrated analysis of ChIP-seq and ATAC-seq data

The downstream analysis of seedling and flower datasets were conducted using the respective set of combined binding sites described above. The tool “featureCounts” (v. 2.0.2) [48] with default parameters was used to obtain raw mapped read counts for the combined binding site regions in each ATAC-seq and/or ChIP-seq samplesRaw mapped reads counts were normalised using RPKM. The library sizes required for RPKM normalisation were calculated using the command “samtools view -c” in a custom script based on the processed mapped reads (data S5).

When needed, differences in chromatin accessibility between different genotypes and time points was quantified with the tool DESeq2 (v. 1.38.3) [49] on the raw read counts of the combined regions. All samples were analysed jointly with the function “DEseq()” using default parameters except parameter *fitType* which was set to”local”. The results of the Differential Accessibility analysis are in data S5.

In order to associate AP1-bound genomic regions with genes which are potentially regulated by AP1 WT/tet-mutant, binding sites 3 kb upstream of the gene, within a gene body to 1 kb downstream of the gene were linked to the respective gene(s).

### RNA-seq analysis

The basic processing of RNA-seq data was conducted in a manner similar to the analysis of ATAC-seq and ChIP datasets described above, except that STAR (v. 2.7.9a) [50] was used to map reads to the *A. thaliana* (Araport 11) with default parameters and PCR duplicates were not removed during post-alignment processing. Coverage tracks of mapped reads were generated with the command “bamCoverage” of the deeptools package (v. 3.5.1) [46] with the parameters bs 1, –skipNonCoveredRegions, –normalizeUsing CPM. To obtain read count matrices of mapped reads the tool “featureCounts” (v. 2.0.2) [48] with default parameters was used. The raw counts obtained for all datasets were normalised using RPKM. The library sizes required for RPKM normalisation were calculated using the command “samtools view -c” in a custom script based on the processed aligned reads. Raw and normalised counts were combined into one dataset (data S6). DEseq2 was used to quantify differences in gene expression between different time points and genotypes using the function “DEseq()” with default parameters using the raw read counts (data S6).

### MNase-seq analysis

Similar to ChIP-seq and ATAC-seq, at first sequencing quality was assessed. Next, reads were mapped to the A. thaliana genome (TAIR 10) using Bowtie2 [42] (v. 2.4.4) with default parameters. Subsequently, samtools [44] (v. 1.13) was used to convert aligned reads (.sam) into.bam format, sort reads by coordinates, remove unmapped reads, remove reads failing platform quality checks, remove reads with a mapping quality < 40. For further analysis, biological replicates of mapped reads were merged using “samtools merge”. DANPOS3 [51] (Python3 version of danpos-2.2.2) was used to perform peak calling and read coverage file generation with default parameters. The called peaks (.smooth.positions.xls) and generated read coverage files (.wig) were transformed into.narrowPeak and.bigwig files respectively using a custom script. Read coverage heatmaps above AP1 binding sites were generated with CoverageView (v. 1.44.0) in R.

### Definition of AP1 binding site categories

To analyse the role of AP1 as a pioneer factor, the merged set of potential AP1 BSs in seedlings was grouped into nine categories based on the significant (FDR < 0.1) binding of AP1 at 2 h uninduced (6,219 sites), the chromatin accessibility of these regions (ATAC-seq 2 h uninduced) and the change of chromatin accessibility from 2 h uninduced to 48 HAI samples. In particular, only pooled AP1^WT^ bound regions that were called significant (FDR < 0.1). Those 6,219 sites were divided into closed (“all_closed”, 2,194 sites) and open (“all_open”, 4,025 sites) chromatin regions based on their chromatin accessibility (RPKM) at 2 h uninduced samples. The threshold used to separate closed from open chromatin was obtained using HMMRATAC [52]. We applied HMMRATAC with default parameters to the combined mapped reads file of the ATAC-seq 2 h uninduced samples to identify nucleosome free regions, and nucleosome containing regions (data S4). The chromatin accessibility threshold (40.98 RPKM) was chosen as the midpoint between the median chromatin accessibility of regions overlapping the detected nucleosome free regions (30.17 RPKM) and the regions overlapping the detected nucleosome containing regions (51.79 RPKM). Both broad categories (“all_closed” and “all_open”) were further divided based on the increase, steady or decrease of chromatin accessibility from 2 h uninduced to 48 HAI ATAC-seq samples using the thresholds: 1) FC > 1.2 & *p*-value < 0.1 for increasing regions (“wt_closed_incr” and “wt_open_incr”), 2) log2FC < 0.8 & *p*-value < 0.1 for decreasing (“wt_closed_decr” and “wt_open_decr”), 3) 0.8 < FC > 1.2 & pvalue > 0.1 for steady (“wt_closed_steady” and “wt_opened_steady”). We used DESEQ2 to calculate FCs and *p*-values between samples (as described above). All regions not classified as increasing, steady or decreasing in chromatin accessibility were assigned to the category “other”.

In a similar fashion AP1^tet^ binding sites were grouped into the categories “tet_all_closed “ and “tet_all_open” based on their chromatin accessibility in 2 h uninduced samples using the same threshold (= 40.98 RPKM) as described above.

### Definition of AP1WT and AP1tet specific binding sites in seedlings

The AP1^WT^ and AP1^tet^-specific regions in seedlings are defined based on the log2 fold change between AP1^WT^ and AP1^tet^ ChIP-seq counts (RPKM). In particular, AP1^WT^ specific regions are the top n (*n* = 200 or 1,000) regions with the highest log2 fold change compared to AP1^tet^. The set of random genomic regions used in Fig. 4 d consists of 1,000 regions with a length of 400 bp each.

### Distance-specific enrichment of SELEX-seq peaks

The significance of enrichment of AP1 SELEX-seq peak distances (Figs. 2e and 3e) within the top n (based on peak score) AP1 binding sites compared with the distance of AP1 SELEX-seq peaks in the whole genome was measured using a hypergeometric test. The test was performed in windows of size 20 bp. A SELEX-seq peak was considered within an AP1^WT^ or AP1^tet^ ChIP-seq when it was within a distance of 200 bp of the centre of the AP1-bound genomic region.

### Statistical analysis and visualisation

All plots were generated with the R programming language (v. 4.2.2) using the graphical visualisation packages ggplot2 (v. 3.4.1) and ComplexHeatmap (v. 2.14.0) [53]. Statistical tests were conducted with base R functions. The distribution of data points is represented by box-plots. The boxes extend from the lower to upper quartile values of the data, with a line at the median. The whiskers extend to 1.5 of the interquartile range. GO-term enrichment analysis was performed with the package topGO (v. 2.46.0) [54] with the parameters nodeSize = 40, algor = classic, test_statistics = Fisher_Exact.

## Supplementary Information

Additional file 1: Fig. S1. Protein Western blot and DNA binding of AP1 wildtype and tetramerisation mutants *in vitro*. Fig. S2. Determination of the dissociation constant. Fig. S3. Complementation analysis of AP1^WT^ and tetramerisation deficient genotypes Fig. S4. AP1^WT^ and AP1^tet^ interact with chromatin remodelers. Fig. S5. ChIP-seq binding comparison between AP1WT and mutants. Fig. S6. Experimental overview Fig. S7. Quality control and summary of seedling datasets Fig. S8. Binding sites of AP1^WT^become more open in seedlings and in floral tissues. Fig. S9. Effects of AP1 pioneer activity in seedlings and flowers Binding of AP1 to nucleosomes. Fig. S10. MNase-seq and AP1-H3 sequential ChIP-seq data. Fig. S11. Binding of AP1 to nucleosomes at different CArG box insertion positions. Fig. S12. AP1 binds to nucleosomes.

Additional file 2: Table S1. IP-MS results on AP1^WT^-GFP and AP1^tet^-GFP inflorescences.

Additional file 3: Table S2.Results of ChIP-seq peak calling for inflorescence and seedling datasets.

Additional file 4: Table S3. Results of peak calling of SELEX-seq data in the genome.

Additional file 5: Table S4. Results of ATAC-seq peak calling using HMMRATAC.

Additional file 6: Table S5. Combined ATAC-seq, ChIP-seq, and DNase-seq analysis of pooled AP1^WT^ and AP1^tet^ binding sites in seedlings.

Additional file 7: Table S6. RNA-seq analysis of AP1^WT^ and AP1^tet^ in seedlings.

Additional file 8: Table S7. List of primer sequences.

Additional file 9: Table S8. Complementation tables for AP1^WT^-GFP, AP1^tet^-GFP and AP1^dm^-GFP.

Additional file 10: Fig. S1a. Uncropped Western Blot image of various AP1 proteins.

Additional file 11. Review history.

## Data Availability

The raw RNA-seq, ChIP-seq, seq-ChIP-seq, MNase,seq, and ATAC-seq data used and generated in this study have been deposited in the GEO database under accession number GSE227951 [55]. The SELEX-seq data are deposited in the GEO database under accession number GSE230171 [56]. The mass spectrometry proteomics data have been deposited to the ProteomeXchange Consortium via the PRIDE partner repository with the dataset identifier PXD041159 [57]. The other publicly available data used is accessible via GEO. SVP ChIP-seq (GSE54881) [58]; SOC1 ChIP-seq (GSE45846) [59]; AP3 and PI ChIP-seq GSE38358 [60]; AP1 ChIP-seq at day 2, day 4 and day 8 of floral meristems (GSE46986) [4]; DNase-seq at day 2, day 4 and day 8 after induction of flower development (GSE46894) [4]; Histone H3 ChIP-seq (GSE106942) [21].
