# Supplementary material for: AP1 is a pioneer transcription factor that programmes cell fate through MADS-domain protein tetramerisation
Source: Genome Biol. 2025 Dec 9;26:418. doi: 10.1186/s13059-025-03884-0 (PMC12687491; doi:10.1186/s13059-025-03884-0)
Supplement: Supplementary file 1 — Additional file 1: Fig. S1. Protein Western blot and DNA binding of AP1 wildtype and tetramerisation mutants in vitro. Fig. S2. Determination of the dissociation constant. Fig. S3. Complementation analysis of AP1WT and tetramerisation deficient genotypes Fig. S4. AP1WT and AP1tet interact with chromatin remodelers. Fig. S5. ChIP-seq binding comparison between AP1WT and mutants. Fig. S6. Experimental overview Fig. S7. Quality control and summary of seedling datasets Fig. S8. Binding sites of AP1WTbecome more open in seedlings and in floral tissues. Fig. S9. Effects of AP1 pioneer activity in seedlings and flowers Binding of AP1 to nucleosomes. Fig. S10. MNase-seq and AP1-H3 sequential ChIP-seq data. Fig. S11. Binding of AP1 to nucleosomes at different CArG box insertion positions. Fig. S12. AP1 binds to nucleosomes. [file 13059_2025_3884_MOESM1_ESM.docx]

**Supplementary Figures**

***
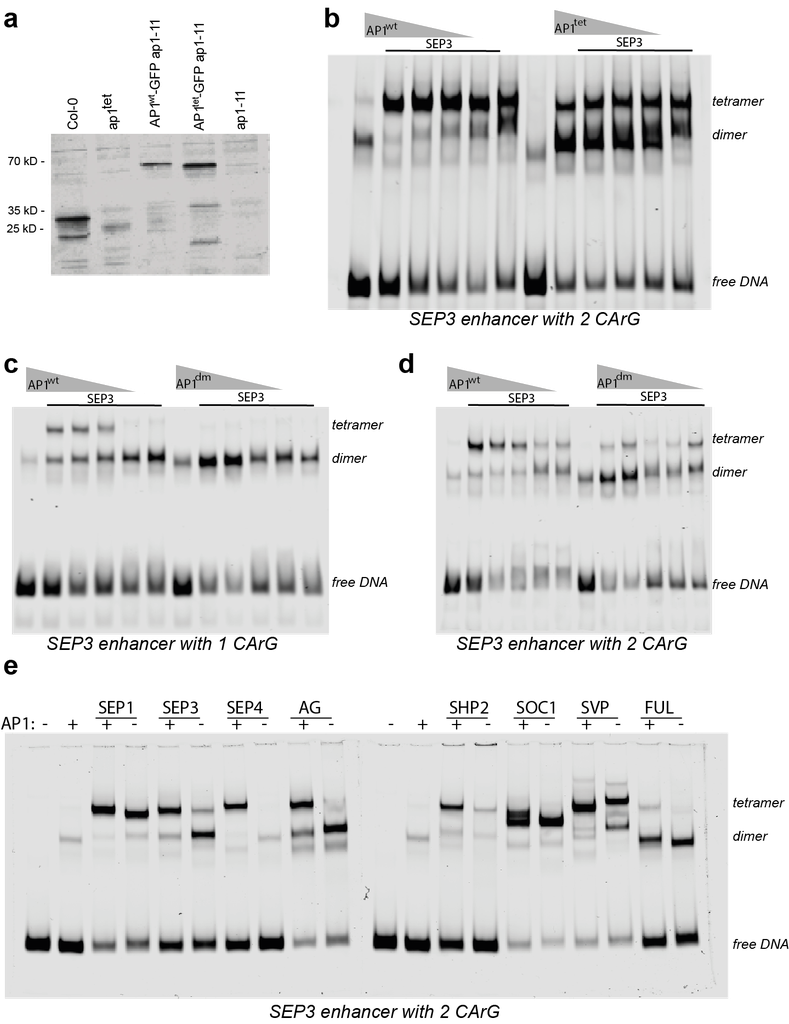
***

Fig. S1 Protein Western blot and DNA binding of AP1 wildtype and tetramerisation mutants *in vitro.* **a** Western blot result showing the AP1 protein level in complementation lines and mutants. **b** EMSA result showing the comparison of DNA binding of AP1^wt^ and AP1^tet^. DNA probe was derived from the SEP3 distal enhancer region containing CArG boxes. **c** EMSA gel image comparing the DNA binding pattern of AP1^wt^ and AP1^dm^. The DNA probe was derived from a SEP3 distal enhancer with one CArG box. **d** EMSA gel picture comparing the DNA binding pattern of AP1^wt^ and AP1^dm^. The DNA probe was derived from a SEP3 distal enhancer region with two CArG boxes. **e** EMSA DNA binding pattern of AP1 showing the interaction of AP1 with other MADS box transcription factors. The DNA probe was derived from a SEP3 distal enhancer region with one CArG box.


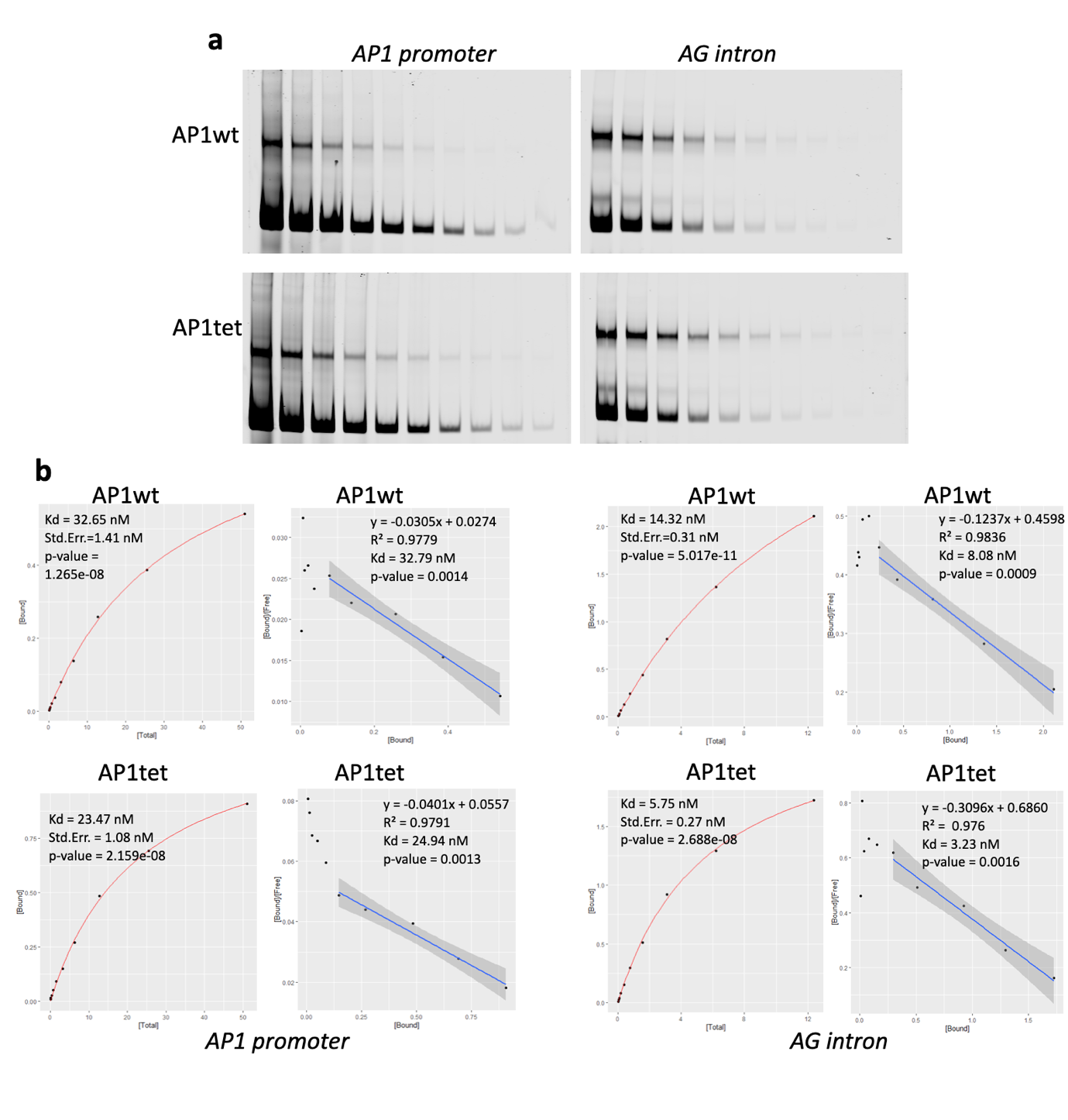


Fig. S2 Determination of the dissociation constant (Kd). **a** EMSA binding reaction of the AP1^wt^ and AP1^tet^ proteins with incremental serial dilutions of the AP1 promoter, AG intron, and SEP3 TSS DNA fragments. **b** Kd estimations with two separate models: a Michaelis-Menten model (Bound ~ Total), red line, and a Scatchard plot (Bound/Free ~ Bound), blue line. Kd estimation for SEP3 TSS DNA fragment was not possible due to a very weak EMSA binding signal.


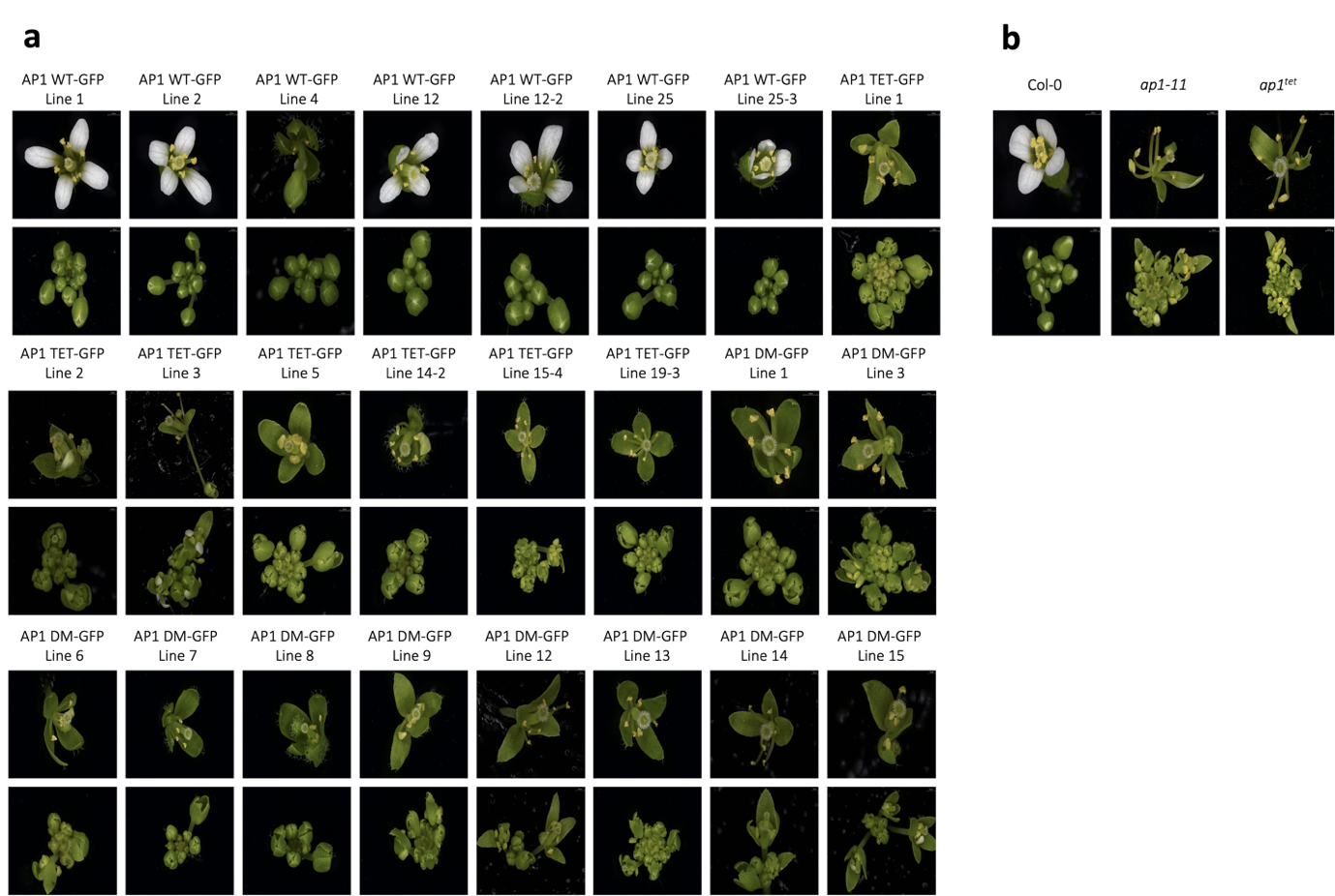


Fig. S3 Complementation analysis of AP1^WT^ and AP1-tetramerisation deficient genotypes. **a** Further phenotypic validation of the AP1^WT^-GFP, AP1^tet^-GFP, and AP1^dm^-GFP transgenic genotypes highlighting the typical phenotypic deficiencies of an AP1 tetramerisation mutant; loss of petal organ initiation and development and loss of sepal organ specification. **b** Phenotypes of inflorescences and mature flowers of the Col-0 ecotype, the ap1-11 mutant, and the CRISPR Cas9-generated ap1^tet^ mutant. For each specimen, flower 3 and inflorescences snipped above flower 5 were imaged (mature flower 3: top image, inflorescence: bottom image).

Fig.S4 Chromatin-associated protein complex partners of AP1^wt^ and AP1^tet^. **a** Volcano plot highlighting the enrichment of chromatin remodelers in AP1 interacting partners based on IP-MS for AP1^WT^-GFP. **b** Volcano plot highlighting the enrichment of chromatin remodelers for AP1^tet^-GFP. **c** Volcano plot comparing the enrichment of chromatin remodeler between AP1^WT^-GFP and AP1^tet^-GFP. Black line: FDR 0.01.

*
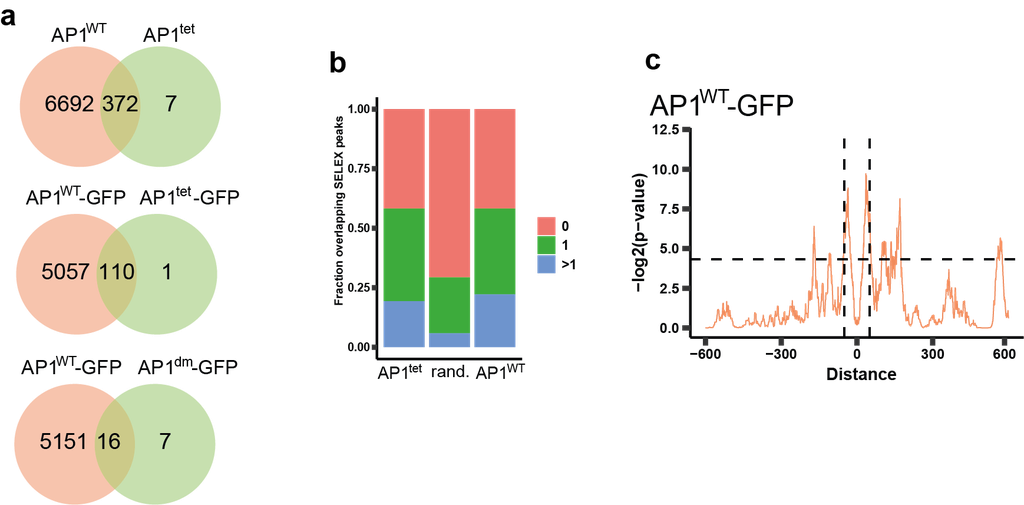
*

Fig. S5 **a** Venn diagram of genes with significant ChIP-seq bound genomic regions of AP1^WT^ vs. AP1^tet^; AP1^WT^-GFP vs. AP1^tet^-GFP and AP1^WT^ vs. AP1^dm^-GFP. **b** Fraction of the top 388 AP1^WT^ and AP1^tet^ binding sites as well as a random set of genomic regions that overlap with zero, one, or more AP1 SELEX-seq peaks. The random set of genomic regions consisted of 388 regions with a length of 400 bp (~ median genomic region length of called peaks) randomly generated, this procedure was repeated 100 times. **c** Enrichment of AP1 SELEX-seq peak distances within the top 388 AP1^WT^-GFP ChIP-seq bound genomic regions. The dashed horizontal line represents a p-value of 0.05. The vertical lines represent the distance +/- 50 bp for visualisation.


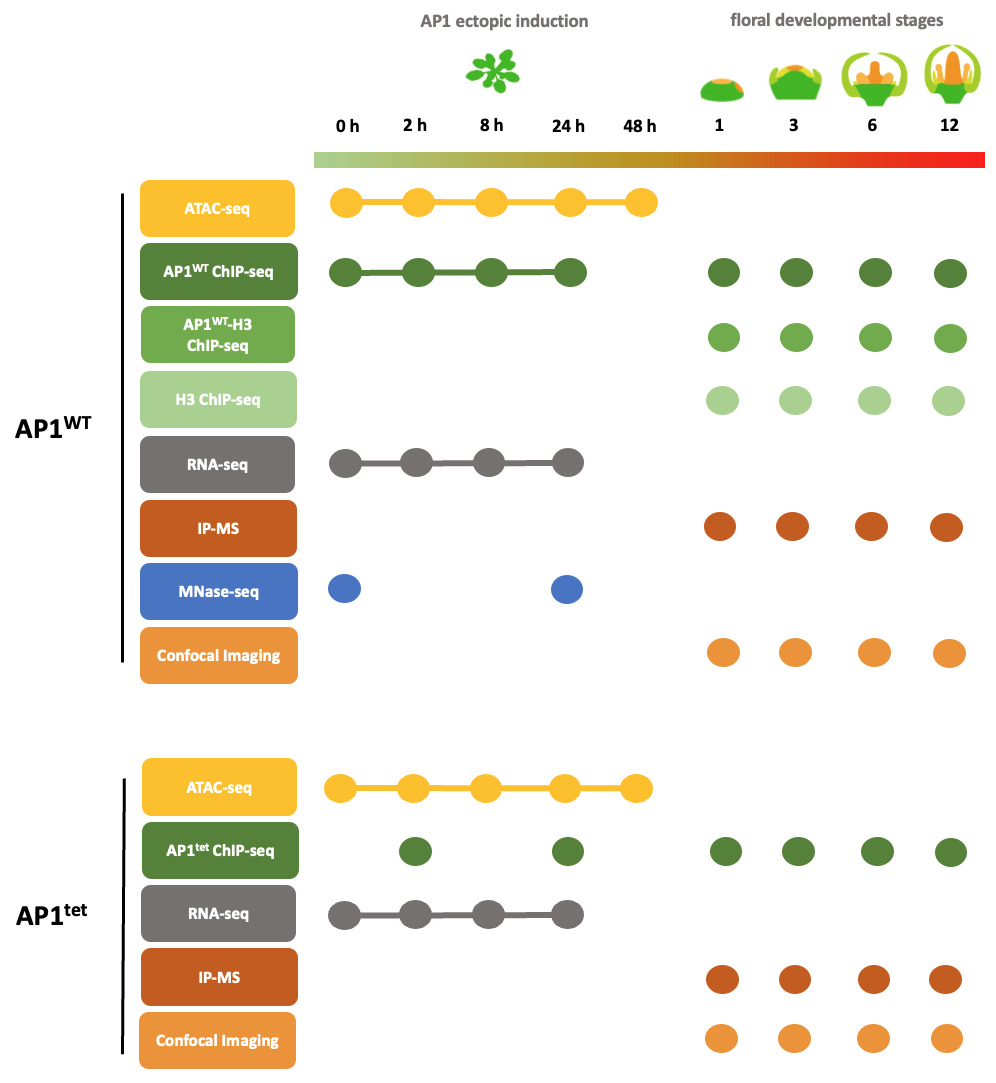


Fig. S6 Experimental overview: Schematic experimental description of ATAC-seq, ChIP-seq, seq-ChIP-seq, RNA-seq, IP-MS, MNase-seq, and confocal live imaging experiments conducted either in seedlings at different time points (connected circles) after ectopic AP1 induction or in entire inflorescences (disconnected circles). These techniques were employed to investigate the role of AP1 as a pioneer transcription factor through tetramerisation.

*
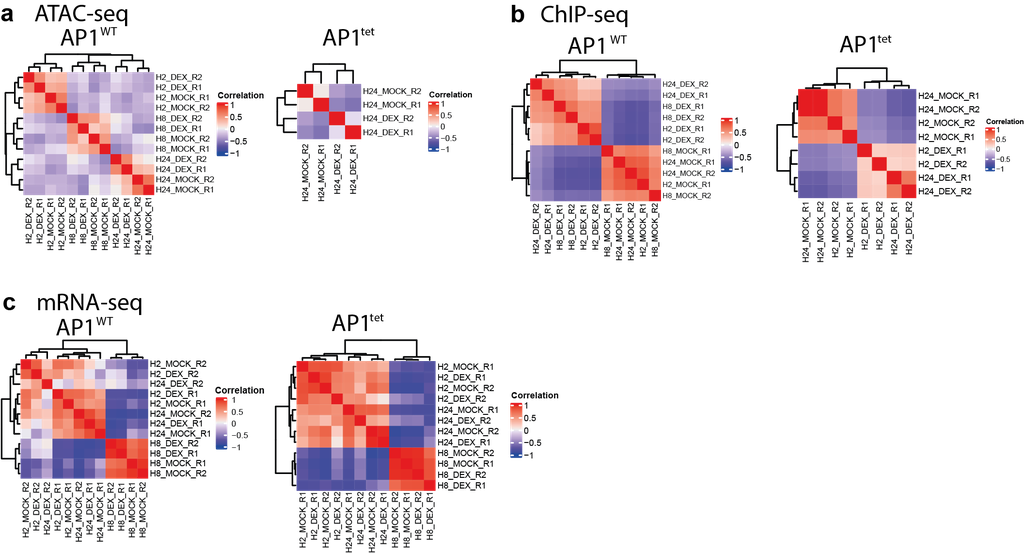
*

Fig. S7 Quality control and summary of seedling datasets. **a-c**. Heat Maps show Pearson correlation of replicates from different time points for DEX and MOCK samples in ATAC-seq, ChIP-seq and RNA-seq datasets for seedlings of different genotypes (AP1^WT^ and AP1^tet^). Correlation was calculated using the relative signal (RPKM normalised counts of samples) with respect to the mean signal for each region.

Fig. S8 Binding sites of AP1^WT^ become more open in seedlings and in floral tissues. **a** Genome browser images showing gene loci of the floral regulators SEP1, SEP2, SHP2, and AP3 which are bound by AP1 and become more open after Dex treatment. The binding of AP1^WT^ is strong at 2 h and 24 h. However, the binding AP1^tet^ is much weaker at 2 h than 24 h in these regions.  **b** Genome browser images showing the binding sites of AP1 in SEP3, AG, and AP1 loci become more open during flower development from 0 day to 8 day after Dex induction.


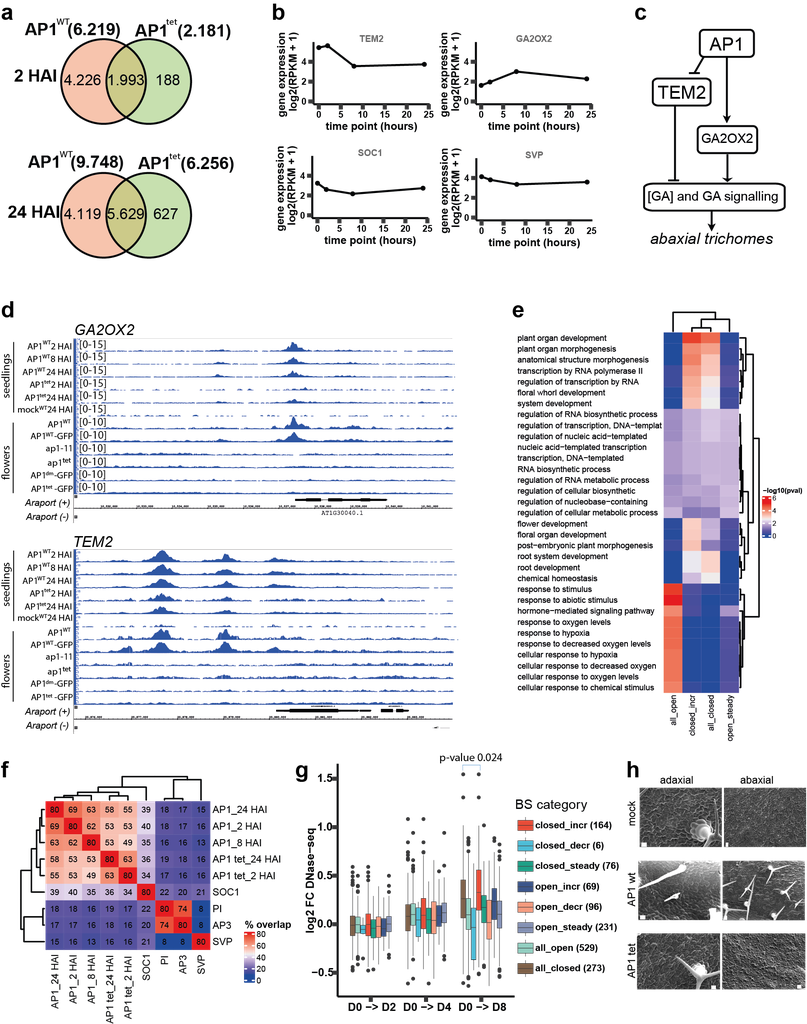


Fig. S9 Effects of AP1 pioneer activity in seedlings and flowers. **a** Venn diagrams of unique and overlapping AP1-bound genomic regions of AP1^WT^ vs AP1^tet^ in the seedling system at 2 HAI and at 24 HAI. **b** Gene expression (log2 (RPKM +1)) for TEM2, GA2OX2, SOC1, and SVP in seedlings at different time points (0h, 2h, 8h and 24h after DEX induction). **c** Proposed model of the regulation of trichome initiation by AP1 in seedlings. **d** ChIP-seq AP1 binding profiles of seedling and flower datasets for GA2OX2 and TEM2 genomic loci. **e** GO-term enrichment analysis for genes in proximity to AP1^WT^ binding sites from different AP1 binding site categories as defined in Fig. 3 f. The enrichment of GO-terms is represented by the significance (-log10(p-value), using Fisher’s Exact test). The genes associated with all combined AP1 binding sites were used as the background for the statistical test. For every binding site category, the top 20 most significantly enriched GO terms (based on p-value) were obtained and the unique set of selected GO terms was used for visualisation in the heat map. **f** The heatmap shows the relative overlap of binding sites between different ChIP-seq datasets of the transcription factors AP1^WT^, AP1^tet^, SOC1, PI, AP3, and SVP in seedlings. Two binding sites are considered overlapping if they overlap by at least 1 bp. The overlap was normalised for binding site length and number of binding sites by using an interval of +/- 200 bp around called peak summits and only considering the top 1,000 called peaks based on peak score. **g** Change in chromatin accessibility from day 0 to days 2, 4, and 8 in flowers for AP1-bound genomic regions of different binding site categories. Chromatin accessibility change is measured as the log2 fold change of RPKM normalised DNase-seq signal between different samples. Only AP1 bound genomic regions from seedlings that overlap with an AP1-bound genomic region in flowers were used (number of binding sites in parentheses). Significance was tested using paired two-sided Mann–Whitney test. The boxes extend from the lower to upper quartile values of the data, with a line at the median. The whiskers extend to 1.5 of the interquartile range. Points indicate outliers. **h** Scanning electron microscopy of epidermal cell. On the adaxial leaf epidermis, the typical leaf-type pavement cell morphology was lost, and stellate trichomes were replaced by unbranched trichomes. On the abaxial side of the leaves, cells reminiscent of ‘giant cell’-like cells were found, and trichomes developed as is typical for early arising sepals, but not for leaves. However, in contrast to sepal trichomes, these abaxial trichomes typically were two-branched.


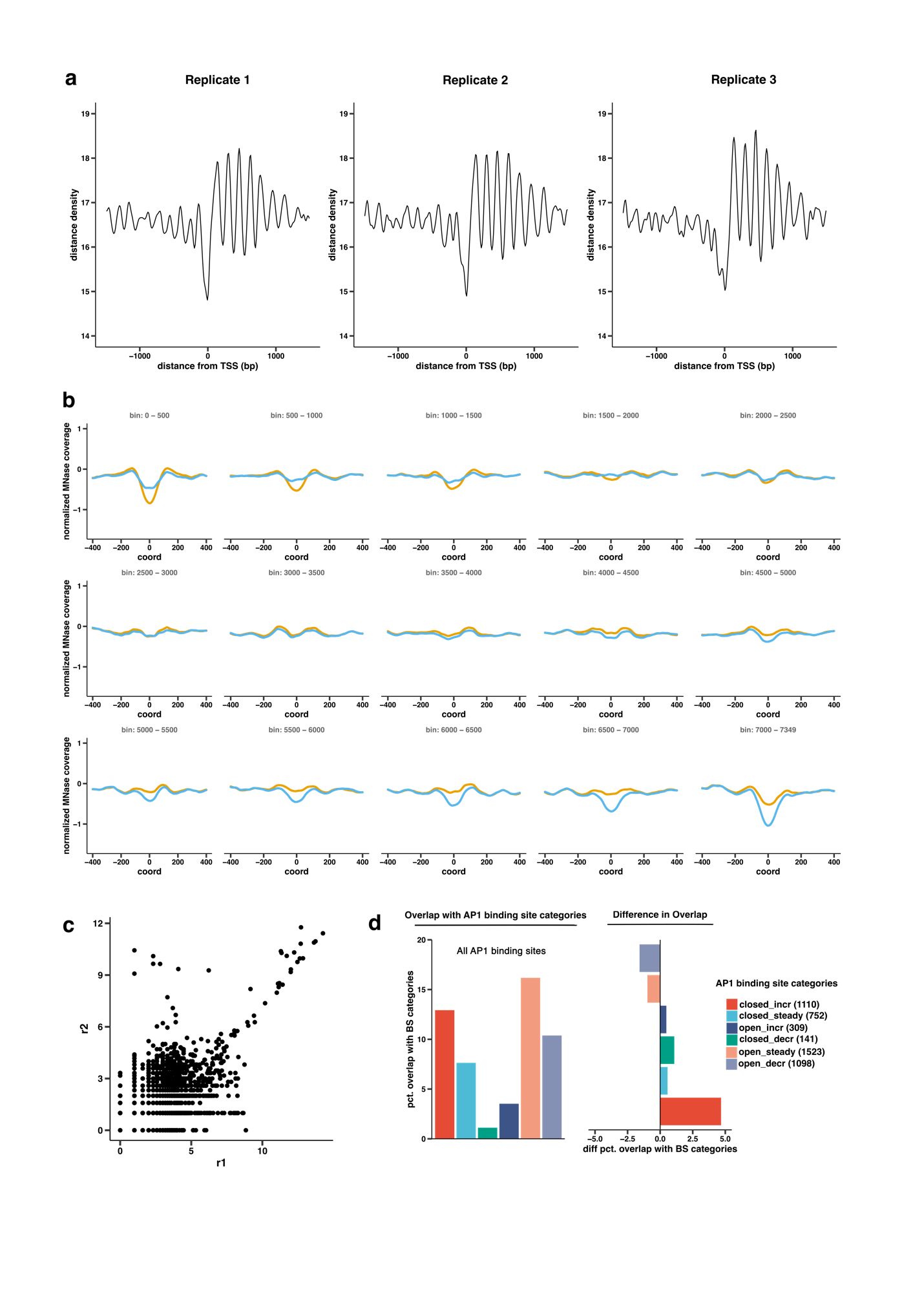


Fig. S10 MNase-seq and AP1-H3 sequential ChIP-seq data. **a** Frequency of MNase-seq called peaks from transcription start site (TSS). **b** Average MNase-seq read count signal above AP1 binding sites for bins containing 500 regions, starting with regions at the top of the heatmap in Fig. 4a. The first groups of regions become more open in the DEX sample over time, while for the last groups of regions this trend is reversed. **c** Log-normalised read counts for merged set of called peaks from sequential AP1-H3 ChIP-seq data for replicates 1 and 2. Pearson correlation coefficient of normalised reads of 0.83 (0.40 for log-normalised reads) indicates high reproducibility. **d** Similar visualisation of overlap as in Fig. 4 c. Overlap of All AP1 binding sites 24 HAI with Ap1 binding sites categories defined in Fig. 3e (left plot) and difference in percentage compared to top 500 AP1 binding sites (group 1) from Fig. 4 a (right plot).

Fig. S11 AP1 binds to nucleosomes. **a** EMSA gel image showing the binding of AP1 to nucleosomes when a CArG box is inserted at Dyad, SHL4-5, or with the original sequence of the Widom 601sequence. **b** EMSA gel image showing the binding of AP1 to nucleosomes when a CArG box is inserted at SHL4-5, SHL1-0 and SHL3-4.


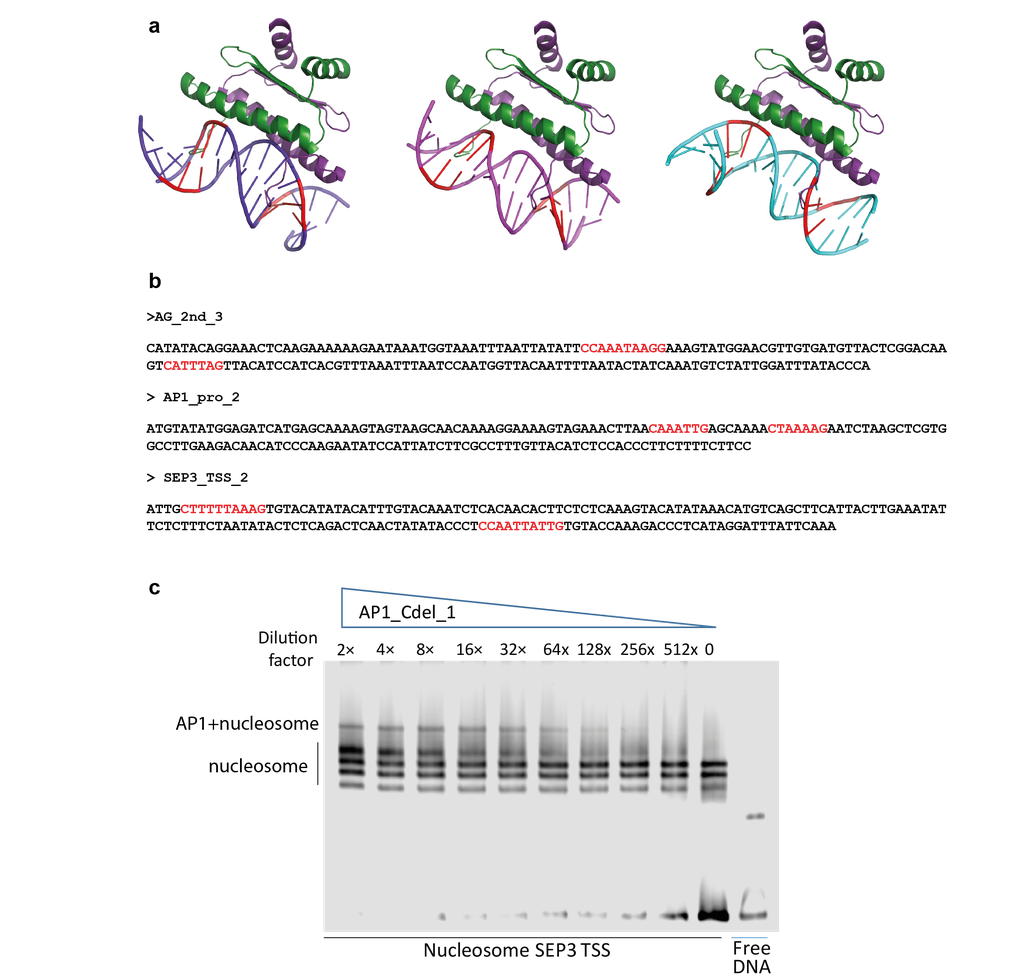


Fig. S12 Binding of AP1 to nucleosomes. **a** Structure modeling of AP1 binding to DNA where DNA-bound structures with MEF2A were used as a DBD template. Red: CC/GG bases. DNA sequences: SHL2-3 is in light purple, DYAD is in dark purple, SHL4-5 is in cyan. **b** Sequence of DNA probes derived from native promoter regions used for nucleosome reconstitution. Potential CArG boxes are highlighted in red. **c** Nucleosomal DNA binding assay with sequential diluted AP1 protein indicating specific binding of AP1 to nucleosomes at SEP3 TSS region.
