# Supplementary figures and images for: AP1 is a pioneer transcription factor that programmes cell fate through MADS-domain protein tetramerisation
Source: Genome Biol. 2025 Dec 9;26:418. doi: 10.1186/s13059-025-03884-0 (PMC12687491; doi:10.1186/s13059-025-03884-0)

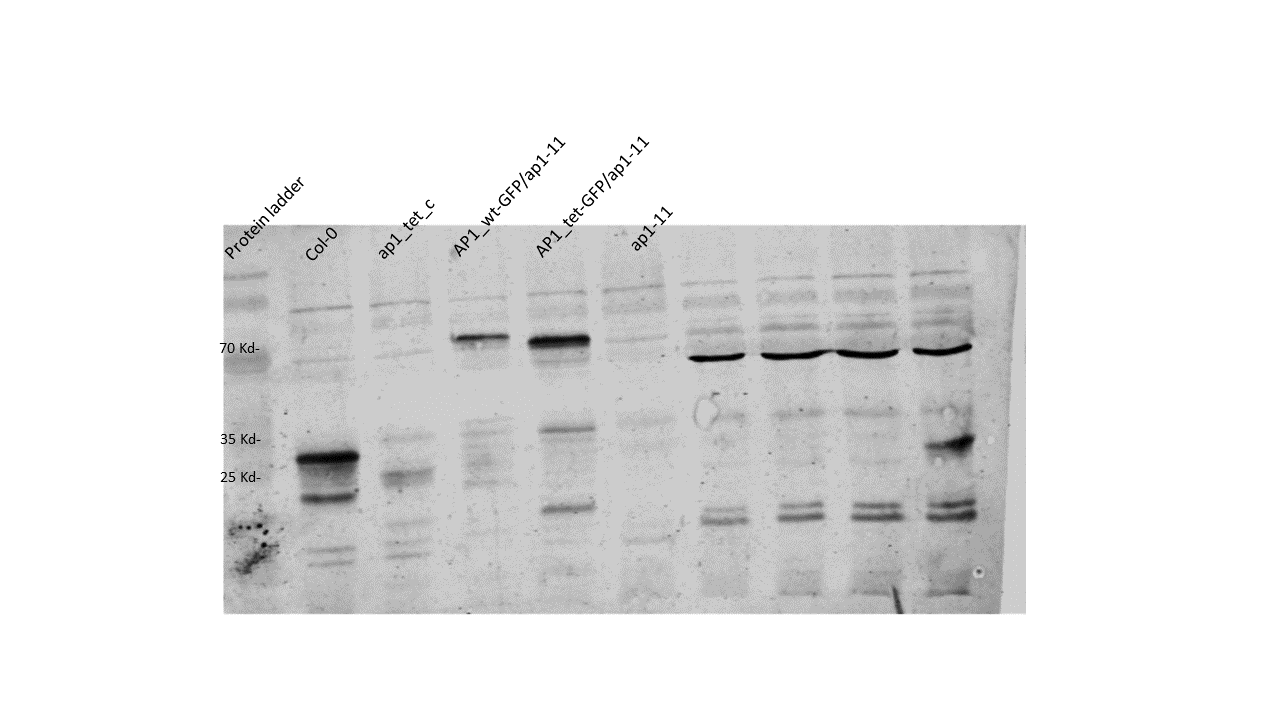

Supplement: Supplementary file 10 — Additional file 10: Fig. S1a. Uncropped Western Blot image of various AP1 proteins. [file 13059_2025_3884_MOESM10_ESM.png]
