# Supplementary material for: AP1 is a pioneer transcription factor that programmes cell fate through MADS-domain protein tetramerisation
Source: Genome Biol. 2025 Dec 9;26:418. doi: 10.1186/s13059-025-03884-0 (PMC12687491; doi:10.1186/s13059-025-03884-0)
Supplement: Supplementary file 11 — Additional file 11. Review history. [file 13059_2025_3884_MOESM11_ESM.docx]

Review History

**First round of review**

**Reviewer 1**

**Were you able to assess all statistics in the manuscript, including the appropriateness of statistical tests used?**

*No, I do not feel adequately qualified to assess the statistics.*

**Comments to author:**

This manuscript entitled "AP1 is a pioneer transcription factor that programmes cell fate through MADS-domain protein tetramerization" by Xu et al describes that AP1 functions as a pioneer TF by facilitating tetramerization. LFY and AP1 are master regulators for floral organ identity genes. LFY has been reported to function as a pioneer TF. Here, the authors reported that AP1 is another pioneer TF in Arabidopsis. By a combination of RNA-seq, ATCA-seq, and ChIP-seq analysis, and in vitro analysis, they showed that the K-domain of AP1 is important for the tetramerization of AP1 and related functions. 
 
The manuscript is well written, methods are described properly, my major concerns are: 
 
1, All the seq data do not have gene annotations. It states the "start" and 'end" of a peak on each chromatin, but it is very difficult for the readers to go through the manuscript to evaluate the consistency of the data, e.g. if the same genes are up or down regulated, what happened to their chromatin structure, and if AP1wt and AP1tet bind to them similarly or differently. 
 
2, The manuscript is lack of independent verification of AP1 as a pioneer TF on at least one or two genes. The seq data suggest that AP1 binds to closed chromatin and induces chromatin opening afterwards, it will be nice to have detailed examination of one or two genes whose chromatins are closed and targeted by AP1 at beginning, and their chromatins are open after certain time of AP1 binding. 
 
3, line 20-50, the data showed that AP1tet affects DNA binding at 2 HAI, but AP1tet binding increased a lot at 24 HAI. How much overlap is there between the AP1wt and AP1tet at 24 HAI? If the genes for petal development such as AP3 and PI are enriched at 2 HAI, but not enriched at 24 HAI in AP1tet, it suggests that tetramerization is important for AP1 function. If AP3 and PI are not enriched at 2 HAI in AP1tet, but enriched at 24 HAI in AP1tet, it suggests that AP1tet affects the binding of AP1 to its targets at the beginning, and eventually it binds to its targets. There are a lot of shared targets between AP1WT and AP1tet at 24 HAI (Fig 3a), and the chromatin accessibility change is only significant at 20%, not at higher percentage. Is there an alternative approach to approve that 20% chromatin accessibility change makes a significant change in its transcription?

**Reviewer 2**

**Were you able to assess all statistics in the manuscript, including the appropriateness of statistical tests used?**

*Yes, and I have assessed the statistics in my report.*

**Comments to author:**

Pioneer transcription factors (TFs) represent a class of TFs that bind to condensed and inaccessible chromatin regions, helping to open the tightly packed DNA and making it accessible for other TFs or other machinery to initiate transcription. The unpackaging role of pioneer transcription factors (TFs) is crucial for cell differentiation, development, or cellular response to stimuli, as it primes the genomic landscape for subsequent gene regulation. Comparing with mammals, the studies about pioneer transcription factors in plants is relatively limited. Only very few of plant pioneer TFs have been reported and characterized. 
 
The paper submitted by Xu et al. very comprehensively explored the function of pioneer transcription factor AP1 in Arabidopsis. Their study specifically analyzed the role of protein tetramerization in shaping the function of AP1. The results from this study is solid, supported by both in vivo and in vitro assays of cellular, genetic, biochemical evidences. The writing is logical, clear, and easy to follow. Here are a few comments that could enhance the overall quality of the study. 
 
Major comments: 
 
1 In the IP-MS data of the AP1-WT and AP1-tet, were any chromatin remodeling-related proteins identified? Including such information would be beneficial for future studies as it could shed light on potential interaction partners functioning in chromatin remodeling. 
 
2 In Figure 3e, the authors compare chromatin accessibility between induced and uninduced samples, revealing significant differences. To provide a more direct evidence of the impact of tetramerization on AP1 binding activity, consider incorporating AP1 foot printing analysis at these differential sites. This would demonstrate how the binding activity directly correlates with the tetramerization state. 
 
3 The in vitro assay at figure 4 demonstrates that AP1 exhibits higher affinity towards nucleosomes. Expanding this finding by comparing chromatin accessibility at AP1 binding regions across different developmental stages or tissues, particularly during the formation of floral meristem, could further support the idea that these regions are nucleosome-condensed prior to AP1 binding. This information could be included in or after Fig. 3f. 
 
Minor comment: 
Please specify whether the tetramerization form of AP1 was used in this prediction in Figure 4b. It would be valuable to compare the differences in binding activity between different forms of AP1.

**Reviewer 3**

**Were you able to assess all statistics in the manuscript, including the appropriateness of statistical tests used?**

*Yes, and I have assessed the statistics in my report.*

**Comments to author:**

The paper by Xu et al. focuses on AP1 MADS transcription factor tetramerization and pioneering activity. This is an important topic and, with revisions as outlined below, the data will represent a significant advance.

My main concerns are outlined below

1. The binding of AP1tet to naked DNA should be examined by quantitative EMSA at different loci (i.e. varying the protein concentration and calculating a KD). This is critical for establishing whether tetramerization is required for chromatin association.

Mutating the tetramerization interface has strong effects on AP1 activity in vivo - flowers lack petals and affects interaction with AP3 and PI based on IP-MS. It also affects DNA binding in vitro, especially at the AG locus (naked DNA, Fig. 4e). It is likely that AP1tet cannot bind naked DNA effectively based on both the in vitro and in vitro data (Fig. 2b).

2. Defining characteristics of a pioneer transcription factor that it can bind its cognate binding motif in the context of a nucleosome both in vitro and in vivo (see Zaret Ann. Rev. 2020 for example). The authors do not show in vivo binding of AP1 to nucleosome occupied sites based on MNAse-seq (conducted in the same conditions +/- dex).. This is key data missing from this manuscript that is required to support their claim. Even so, the ChIP-seq and MNase-seq data are cell population averages from many different cell types, such that AP1 could bind in some cell types to a given cis motif, yet this cis motif occupied by a the nucleosome in other cell types. Because of that and additional test for AP1 and the nucleosome simultaneously occupying the same piece of DNA is sequential AP1 and histone ChIP or similar analyses. These data need to be provided to shore up the conclusion that AP1 is a pioneer transcription factor. Finally, Figs. 3e and f are not relevant to the pioneering activity of AP1 as discussed below and should be removed.

a. A key concern here is that the authors use ATAC seq to test whether AP1 binding sites are nucleosome occupied. This is too coarse a method to assess in vivo pioneer factor activity. The authors should follow the well-established procedures for defining pioneer factor activity i.e. compare ChIP-seq with MNase-seq before and after induction of AP1. A case in point: Figure 3d shows that a window of 100 bp around the AP1 binding sites is devoid of ATAC-seq defined 'closed' chromatin, this suggests plenty of accessible DNA for AP1 to bind its cognate motives even in the 'closed' chromatin state population.

b. Fig. 3 should focus only on wild-type AP1. Fig. 4e shows that AP1tet binds naked DNA very poorly. If naked DNA binding is affected, this version of AP1 is not informative vis-à-vis chromatin binding! Since AP1tet binds very poorly also based on ChIP-seq (Fig. 2b), one would expect poor correlation with binding sites and poor activation/change in chromatin at nearby genes (Fig. 3c and b respectively). Hence, the AP1tet data are not informative in the context of the question being investigated.

c. Last but not least, the authors define pioneer transcription factors as factors that can alter chromatin accessibility. This is not a defining feature of pioneer transcription factors. Opening chromatin by recruiting chromatin regulators is a common feature of all transcription factors. Indeed, pioneer transcription factors often bind and trigger only small local chromatin changes (a few bp, often without recruiting additional factors) to allow binding of non-pioneer partners. These TF partners subsequently, together with the pioneer factor, generate accessible chromatin by recruiting chromatin regulators as assessed by ATAC-seq (Li/Grosschedl G&D2018, Myran/ Drouin NG2018, Sardina/Graf Cell stem Cell 2018). Thus, Figs 3e and f do not speak to pioneering activity. They also report very late events, after 2 day activation, unlike all other date presented in the manuscript. Such late events are likely caused by downstream effects, i.e. may be the consequence of AP1 target gene activation.

3. Figure 4 addresses AP1 binding to nucleosomes in vitro. Overall the evidence here is supportive of AP1's ability to bind nucleosomes in vitro. However, for Fig. 4b the authors need to replace the data shown with ChIP-seq and MNAse-seq, as mentioned above (point 2) and include screenshots for MNAse-seq prior to AP1 binding to show whether the binding site is nucleosome occupied prior to binding or not. Moreover, the data provided does not show that AP1 displaces a nucleosome in Fig. 4e. The reduction in the nucleosome bands upon AP1+SEP3 binding is fully attributable to the shifted band (arrow in Fig. 4e). Please remove this statement. Finally, Fig. 4e shows that AP1 cannot access its binding site in the context of the nucleosome at the AG locus; it can only bind when SEP3 is present. This should be discussed further.

Additional comments:
a. The Widom sequence is considered too strong a nucleosome positioning sequence by many in the pioneer TF field to assess in vitro binding of pioneer factors, it could be that SHL4/5 work well since they are close to the exit of the DNA from the nucleosome? Also the DBD can be placed in many other contexts on the nucleosome, I would think!
b. Use of endogenous sequences is a plus here. However, in all cases the nucleosomes are not that well assembled (they should not have multiple bands). Did the authors us the heat step to isolate only stable nucleosomes for further experiments?
c. The free DNA bands should be included in the picture for Figs. 4d and e.
d. Throughout in all EMSAs it would be best to state the DNA and protein amount used and to calculate KDs, as described in Soufi Cell 2015.

**Authors Response**

**Point-by-point responses to the reviewers’ comments:**

**Reviewer 1**

This manuscript entitled "AP1 is a pioneer transcription factor that programmes cell fate through MADS-domain protein tetramerization" by Xu et al describes that AP1 functions as a pioneer TF by facilitating tetramerization. LFY and AP1 are master regulators for floral organ identity genes. LFY has been reported to function as a pioneer TF. Here, the authors reported that AP1 is another pioneer TF in Arabidopsis. By a combination of RNA-seq, ATAC-seq, and ChIP-seq analysis, and in vitro analysis, they showed that the K-domain of AP1 is important for the tetramerization of AP1 and related functions. The manuscript is well written, methods are described properly, my major concerns are:

1. All the seq data do not have gene annotations. It states the "start" and 'end" of a peak on each chromatin, but it is very difficult for the readers to go through the manuscript to evaluate the consistency of the data, e.g. if the same genes are up or down regulated, what happened to their chromatin structure, and if AP1wt and AP1tet bind to them similarly or differently.

***Reply:*** *Thanks for the suggestion. Genomic regions have been annotated with gene names and are now part of the supplemental information, making it easier for the reader to follow the manuscript (Fig 5d and Fig.S11). We also added gene names to the peak regions in Fig.2b, so readers can now go through the figures with more clarity. As suggested by the reviewer, we included figures showing the binding of AP1 and AP1^tet^ to genomic loci of several flower master regulators and changes in their chromatin status and transcriptional activity before and after binding to help the readers evaluate the consistency of the data (Fig.5g and Fig.S11a).*

2. The manuscript is lack of independent verification of AP1 as a pioneer TF on at least one or two genes. The seq data suggest that AP1 binds to closed chromatin and induces chromatin opening afterwards, it will be nice to have detailed examination of one or two genes whose chromatins are closed and targeted by AP1 at beginning, and their chromatins are open after certain time of AP1 binding.

***Reply:*** *We agree with reviewer 1 that it would be good to exemplify specific genomic regions where AP1 acts as a pioneer transcription factor. We have now included Fig. 5d to show the pioneer activity of AP1 on floral master regulator genes AG, AP1 and SEP3 and Fig.S11 to show further examples.*

3. line 20-50, the data showed that AP1tet affects DNA binding at 2 HAI, but AP1tet binding increased a lot at 24 HAI. How much overlap is there between the AP1wt and AP1tet at 24 HAI? If the genes for petal development such as AP3 and PI are enriched at 2 HAI, but not enriched at 24 HAI in AP1tet, it suggests that tetramerization is important for AP1 function. If AP3 and PI are not enriched at 2 HAI in AP1tet, but enriched at 24 HAI in AP1tet, it suggests that AP1tet affects the binding of AP1 to its targets at the beginning, and eventually it binds to its targets. There are a lot of shared targets between AP1WT and AP1tet at 24 HAI (Fig 3a), and the chromatin accessibility change is only significant at 20%, not at higher percentage. Is there an alternative approach to approve that 20% chromatin accessibility change makes a significant change in its transcription?

***Reply:*** *Thank you for the insightful comment, which helped us refine and clarify our findings in the revised manuscript: As Reviewer 1 noted, the difference in chromatin accessibility between AP1wt and AP1tet is significant only in strongly inaccessible regions. We would like to clarify that the “20%” refers to the rank of chromatin accessibility based on ATAC-seq signal, not a 20% change in accessibility. We have now clarified this in the main text and figure legends. We would also like to note that there are some inherent technical differences in the ATAC-seq and ChIP-seq methods: different from ChIP-seq, ATAC-seq provides a global view across all cells, potentially averaging out signals from cells where AP1 actively binds to a specific genomic regions and cells where it does not. Given the different properties, we consider using ‘ranks’ as the most appropriate method of choice for data integration.*

*Following the reviewer’s suggestion, we examined AP3 as an example locus. The AP3 TSS is located in a relatively closed chromatin region (low ATAC-seq signal; see Fig. S11a). AP1wt binds strongly to the AP3 promoter already at 2 HAI, whereas AP1tet shows low binding at 2 HAI but increased binding at 24 HAI. This suggests that tetramerization facilitates AP1 binding to closed chromatin at early stages when protein levels are lower, while AP1tet dimers achieve higher binding later as protein levels rise. We did not include PI in our analysis, as it was not strongly enriched in ChIP-seq data. Fig. 3a shows the overlap of AP1^WT^ and AP1^tet^ at 2 HAI and 24 HAI across different chromatin accessibility contexts. A broader binding site overview is now provided in Fig. S5A.*

**Reviewer 2**

Pioneer transcription factors (TFs) represent a class of TFs that bind to condensed and inaccessible chromatin regions, helping to open the tightly packed DNA and making it accessible for other TFs or other machinery to initiate transcription. The unpackaging role of pioneer transcription factors (TFs) is crucial for cell differentiation, development, or cellular response to stimuli, as it primes the genomic landscape for subsequent gene regulation. Comparing with mammals, the studies about pioneer transcription factors in plants is relatively limited. Only very few of plant pioneer TFs have been reported and characterized.
The paper submitted by Xu et al. very comprehensively explored the function of pioneer transcription factor AP1 in Arabidopsis. Their study specifically analyzed the role of protein tetramerization in shaping the function of AP1. The results from this study is solid, supported by both in vivo and in vitro assays of cellular, genetic, biochemical evidences. The writing is logical, clear, and easy to follow. Here are a few comments that could enhance the overall quality of the study.

Major comments:

1. In the IP-MS data of the AP1-WT and AP1-tet, were any chromatin remodeling-related proteins identified? Including such information would be beneficial for future studies as it could shed light on potential interaction partners functioning in chromatin remodeling.

***Reply:*** *Thanks for the question. Yes, according to the IP-MS data, AP1^WT^ interacts with chromatin remodelling factors. We further observed a reduction in chromatin remodeler interactions in the AP1^tet^ mutant when comparing the AP1^tet^ eluate vs the AP1^tet^ input samples. We mention the interactions in the first result section of the main text (section “Tetramerisation is required for AP1 function in vivo”) and further highlighted such interactions in a new supplementary figure; Fig. S9. For example, the ATP-dependent nucleosome remodelers BRAHMA (BRM) and SPLAYED (SYD) that alter nucleosome positioning on DNA were identified, confirming previous findings from our lab and others (Smaczniak et al. PNAS 2012; Wu et al. PNAS 2012). As their levels of enrichment are lower in the AP1 TET mutant IP-MS-data, this observation indicates that AP1 tetramerization can enhance the recruitment of chromatin remodelers to facilitate chromatin opening.*

2. In Figure 3e, the authors compare chromatin accessibility between induced and uninduced samples, revealing significant differences. To provide a more direct evidence of the impact of tetramerization on AP1 binding activity, consider incorporating AP1 foot printing analysis at these differential sites. This would demonstrate how the binding activity directly correlates with the tetramerization state.

***Reply:*** *We agree with reviewer 2 that an evaluation of tetramerization at AP1 bound genomic regions supports our claim that a loss of tetramerization ability reduces the AP1 occupancy and therefore the ensuing pioneer activity. In the original submitted manuscript, Fig. 3d addresses this point by combining SELEX-seq and ChIP-seq data (i.e. preferred AP1 binding site spacing indicative of binding by tetramers), thus essentially following this line of thinking. Also, it is recommended to have more than 200 million ATAC-seq reads mapped to the genome in order to obtain reasonable results from footprinting analysis with various tools (see review. Yan et al. Genome Biology 2020), but unfortunately, the sequencing depth of our ATAC-seq libraries is currently not high enough (~50 Mio mapped reads). ATAC-seq footprinting nevertheless provides a very useful suggestion for our future research.*

3. The in vitro assay at figure 4 demonstrates that AP1 exhibits higher affinity towards nucleosomes. Expanding this finding by comparing chromatin accessibility at AP1 binding regions across different developmental stages or tissues, particularly during the formation of floral meristem, could further support the idea that these regions are nucleosome-condensed prior to AP1 binding. This information could be included in or after Fig. 3f.

***Reply:*** *Thanks for the suggestion. We have now provided information on the chromatin status for the genomic regions used for Figure 4 (now Fig. 5) using our DNase-seq data (Pajoro et al., Genome Biology 2014) from flowers. We can see that AP1 binding to these genomic regions is found both in flowers and seedlings, and the chromatin becomes more open from day 0 to day 8 after flower induction, which indeed puts the in vitro results into a biological context. We included this information in Fig.S11b.*

Minor comment: Please specify whether the tetramerization form of AP1 was used in this prediction in Figure 4b. It would be valuable to compare the differences in binding activity between different forms of AP1.

***Reply:*** *Thanks for the good suggestion. We are prefacing our answer by stating that as we added an additional figure (now Fig.4), the previous Fig. 4 has been shifted to Fig.5. Thus, the structure we used for modelling in Fig. 5b is the DNA-bound structure for MEF2A, a mammalian homolog of AP1. Currently, the crystal structure of a DNA-bound AP1 MADS-domain is not available. We mentioned this information in the main text pertaining to Fig. 5 and have now also mentioned it in the figure legend of Fig. 5.*

**Reviewer 3**

The paper by Xu et al. focuses on AP1 MADS transcription factor tetramerization and pioneering activity. This is an important topic and, with revisions as outlined below, the data will represent a significant advance.

My main concerns are outlined below

1. The binding of AP1tet to naked DNA should be examined by quantitative EMSA at different loci (i.e. varying the protein concentration and calculating a KD). This is critical for establishing whether tetramerization is required for chromatin association.

Mutating the tetramerization interface has strong effects on AP1 activity in vivo - flowers lack petals and affects interaction with AP3 and PI based on IP-MS. It also affects DNA binding in vitro, especially at the AG locus (naked DNA, Fig. 4e). It is likely that AP1tet cannot bind naked DNA effectively based on both the in vitro and in vitro data (Fig. 2b).

***Reply:*** *Thanks for the suggestion. We tested the binding of AP1^tet^ to ‘naked’ DNA using the probes in Fig. 5c (previously Fig. 4c) by quantitative EMSA. We varied the DNA concentration and calculated a Kd for each probe (Fig.S10, see also below). We found that AP1^tet^ and AP1^WT^ had similar DNA-binding levels (Fig.S10.a). Unexpectedly, AP1tet binds even more strongly than AP1^WT^ to native DNA with a lower Kd number for both probe AP1 promoter and probe AG intron (Fig.S10.b). In the case of SEP3_TSS, we did not see the binding of AP1^WT^ (Fig.S10.a) in this experimental condition, so we did not calculate a Kd for it. But when we used a higher concentration of AP1wt, we did see bindings on SEP3_TSS (now Fig 5.c) with multiple bands. In conclusion, tetramerization is not required for general DNA-binding, but is needed for accessing DNA-binding sites in an in vivo chromatin context.*

*Mutating tetramerization did affect AP1 activity in vivo, based on the strong phenotype of the ap1^tet^ mutant. We did see less binding of AP1^tet^ in what was previously Fig.4e, but this might be due to technical limitations (AP1^tet^ protein levels when expressed in E. coli). Given the reviewer comments and the technical challenges, we decided to omit what was previously Fig. 4e from the revised manuscript.*

New Fig. S10, see Additional File 1 for details.

2. Defining characteristics of a pioneer transcription factor that it can bind its cognate binding motif in the context of a nucleosome both in vitro and in vivo (see Zaret Ann. Rev. 2020 for example). The authors do not show in vivo binding of AP1 to nucleosome occupied sites based on MNAse-seq (conducted in the same conditions +/- dex).. This is key data missing from this manuscript that is required to support their claim. Even so, the ChIP-seq and MNase-seq data are cell population averages from many different cell types, such that AP1 could bind in some cell types to a given cis motif, yet this cis motif occupied by a the nucleosome in other cell types. Because of that and additional test for AP1 and the nucleosome simultaneously occupying the same piece of DNA is sequential AP1 and histone ChIP or similar analyses. These data need to be provided to shore up the conclusion that AP1 is a pioneer transcription factor. Finally, Figs. 3e and f are not relevant to the
pioneering activity of AP1 as discussed below and should be removed.

***Reply:*** *Thanks for the helpful suggestion. As mentioned by reviewer 3, MNase-seq is a powerful alternative method to ATAC-seq which allows for a more fine-grained interrogation of the chromatin accessibility landscape. Consequently, we performed MNase-seq experiments in both +/- dex conditions 24 HAI in AP1-GR expressing seedlings. We present the results in newly added Fig. 4 in Fig. 4a, b and c. We found that the MNase-seq signal overlaps with AP1 ChIP-seq signals from the same tissue, which indicates AP1 binds to nucleosomes in planta. Additionally, after induction, the MNase-seq signals decreased near peak summits of AP1-bound genomic regions (Fig. 4a and b), which supports its role as a pioneer transcription factor. Thus, as the MNase-seq signal decreased near peak summits of AP1-bound genomic regions post dex induction (Fig. 4a and b), it supports the claim that AP1 is a pioneer transcription factor.*

*We also appreciate the reviewers suggestion to corroborate our MNase-seq data with sequential ChIP-seq (seq-ChIP-seq) data. Using native inflorescence tissue we thus generated seq-ChIP-seq data targeting AP1 and histone 3 (H3). The corresponding results are provided in Fig. 4d and e. We defined a set of high-confidence AP1 pioneer regions based on an overlap of concurrent binding sites between our previously generated AP1 WT ChIP-seq dataset, our newly generated AP1/H3 seq-ChIP-seq dataset, and a previously published H3 ChIP-seq dataset (also from native inflorescence tissue). The overlap of these putative AP1 pioneer regions have been included in Fig. 4d. We additionally generated genome browser plots for direct visualisation of some of these shared binding sites to exemplify overlapping peaks. These can now be found in Fig. 4e. Both the MNase-seq and seq-ChIP-seq experiments, as suggested by the reviewer, further confirmed AP1’s potential to access its binding sites and trigger chromatin opening in vivo.*

*We would like to clarify that Fig. 3a and b also provide supportive evidence for the in vivo activity of AP1 in a chromatin context. To clarify this point, we improved the explanation for these figures in the main text. In Fig. 3e, we provide evidence that AP1 can alter the chromatin state of its binding sites following induction in non-floral tissues. Specifically, AP1-bound regions were categorized based on changes in chromatin accessibility—remaining steady, becoming more open, or becoming more closed—after AP1 binding. This categorization provides important insights into the complexity and diversity of AP1’s effects on chromatin dynamics.*

*Fig. 3f provides essential information on the histone modification landscape at AP1 binding sites, categorized as in Fig. 3e. This analysis helps us better understand the interaction between histone modifications and AP1 binding. Notably, AP1 appears to promote chromatin closure at regions marked by either H3K27me3 or H3K4me1, which is intriguing. Furthermore, when AP1tet is induced in non-floral tissues, it is not enriched in chromatin marked by H3K27me3 but can still associate with open chromatin regions enriched in active marks such as H3K9ac and H3K27ac.*

a. A key concern here is that the authors use ATAC seq to test whether AP1 binding sites are nucleosome occupied. This is too coarse a method to assess in vivo pioneer factor activity. The authors should follow the well-established procedures for defining pioneer factor activity i.e. compare ChIP-seq with MNase-seq before and after induction of AP1. A case in point: Figure 3d shows that a window of 100 bp around the AP1 binding sites is devoid of ATAC-seq defined 'closed' chromatin, this suggests plenty of accessible DNA for AP1 to bind its cognate motives even in the 'closed' chromatin state population.

***Reply:*** *We have followed the reviewer’s suggestion to generate MNase-seq data as mentioned above. However, the reviewer misunderstood Fig. 3d. To prevent any further misunderstanding on behalf of the reader, we have added more information in the legend. What is displayed in Fig. 3d are preferred AP1 binding site distances as defined by SELEX-seq in AP1-bound genomic regions that were classified according to their overall chromatin accessibility (accessibility window). The preferred ~50 bp spacing between individual AP1 binding elements (aka CArG boxes) is a feature only found in closed chromatin (accessibility ranks low 0.0-0.2), while in open chromatin AP1 loses this spacing preference. We interpret this finding in the discussion of the manuscript that simultaneous DNA-binding of two AP1-binding elements assists in accessing DNA-binding sites in a more ‘closed’ in vivo chromatin context. In the future, it will be interesting to perform further in vitro and in vivo assays to develop more in-depth biochemical models on how cooperative DNA-binding activities of AP1 and its partners can enhance nucleosome binding. Another interesting aspect that remains to be taken into account in future research is the capacity of AP1 to bend DNA around its binding sites.*

b. Fig. 3 should focus only on wild-type AP1. Fig. 4e shows that AP1tet binds naked DNA very poorly. If naked DNA binding is affected, this version of AP1 is not informative vis-à-vis chromatin binding! Since AP1tet binds very poorly also based on ChIP-seq (Fig. 2b), one would expect poor correlation with binding sites and poor activation/change in chromatin at nearby genes (Fig. 3c and b respectively). Hence, the AP1tet data are not informative in the context of the question being investigated.

***Reply:*** *We agree with the reviewer’s observation that AP1tet does not bind to chromatin strongly by ChIP-seq in floral tissues (Fig.2b). However, AP1 binding to DNA is not generally impaired as shown in our new in vitro EMSA assays (please also see our reply to your first comment). Furthermore, AP1^tet^ does bind readily to genomic regions when ectopically expressed at higher levels in seedlings, and it shares many common binding sites with AP1^WT^ detected by ChIP-seq (Fig. 3a). We can see from the screenshots of AP1^tet^ binding peaks that it can readily bind to open chromatin, but its binding to closed chromatin is almost fully abolished (Fig.3g). We also had a similar concern like the reviewer regarding the low levels of AP1^tet^ binding under native conditions in floral tissues. To rule out the possibility of technical issues, we performed multiple ChIP-seq experiments with different AP1^tet^ mutants, but they all gave similar results.*

*In summary, AP1^tet^ can bind to DNA with similar affinities like AP1^wt^ based on our EMSA experiments, and it reaches similar occupancy levels to AP1^wt^ in ChIP-seq when overexpressed at 24 HAI. In the manuscript, we provide several lines of evidence that AP1^WT^ can access its DNA-binding sites in a nucleosomal context, and that this facilitates AP1 recruitment to more closed genomic regions in planta. We also provide evidence that this capacity is at least strongly reduced in case of the AP1^tet^ mutant.*

c. Last but not least, the authors define pioneer transcription factors as factors that can alter chromatin accessibility. This is not a defining feature of pioneer transcription factors. Opening chromatin by recruiting chromatin regulators is a common feature of all transcription factors. Indeed, pioneer transcription factors often bind and trigger only small local chromatin changes (a few bp, often without recruiting additional factors) to allow binding of non-pioneer partners. These TF partners subsequently, together with the pioneer factor, generate accessible chromatin by recruiting chromatin regulators as assessed by ATAC-seq (Li/Grosschedl G&D2018, Myran/ Drouin NG2018, Sardina/Graf Cell stem Cell 2018). Thus, Figs 3e and f do not speak to pioneering activity. They also report very late events, after 2 day activation, unlike all other date presented in the manuscript. Such late events are likely caused by downstream effects, i.e. may be the consequence of AP1 target
gene activation.

***Reply:*** *We thank the reviewer for raising this interesting point. We would like to point out that we use a more inclusive approach towards understanding AP1 pioneer factor activity beyond its direct binding to DNA-binding sites in a nucleosomal context, thus following recently adopted, more inclusive approaches to understand pioneer activities (see, e.g. Barral and Zaret, 2023). To better validate the AP1 pioneer activities, we now generated MNase-seq and sequential ChIP-seq data to better support our claim that AP1 is a pioneer transcription factor. But as the reviewer also mentioned, the opening of chromatin is an ultimate consequence of pioneer activity, so we prefer to keep Fig. 3e. Fig. 3f indicates AP1’s potential to bind to chromatin in a repressive state, in contrast to AP1tet. We consider this an interesting finding worth reporting in the manuscript, beyond adhering to a narrow focus on biochemically defining AP1 as a pioneer factor.*

*In animal research, 24 h induction in cell lines might be a standard to test pioneer activity. But plants cannot generate cell lines, so we used a chemically inducible seedling system to express a floral pioneer factor. The growing seedlings are more resistant to transcriptional changes than cell lines which are outside of the animal body. Accordingly, the number of DEGs at the early timepoints is very low. Although at 24 hours we already see changes in chromatin (Fig 3. b), 48 h is reasonable to use in plants. In another recently published paper about LFY pioneer activity in Arabidopsis, even constitutive overexpression was used (Lai et al., 2021).*

*The earliest TF targets of AP1 are also MADS-domain TF genes, e.g. SEP3, which forms tetrameric complexes with AP1. As we discuss in the manuscript, given their in vitro DNA-binding behaviour, SEP3/AP1 heterotetramers can be expected to have enhanced capacity to binding to closed chromatin than AP1 homotetramers. Thus, beyond the general technical limitations mentioned above, a feed-forward loop of AP1 and SEP3 may also explain the relatively slow observed effects of AP1 in terms of chromatin opening (AP1 activates SEP3, and directly heterotetramizes with it at protein level, see e.g. Kaufmann, Wellmer et al. Science 2010). Besides, there no other TFs as candidate pioneer factors could be identified in our transcriptomics time-series experiments.*

3. Figure 4 addresses AP1 binding to nucleosomes in vitro. Overall the evidence here is supportive of AP1's ability to bind nucleosomes in vitro. However, for Fig. 4b the authors need to replace the data shown with ChIP-seq and MNAse-seq, as mentioned above (point 2) and include screenshots for MNAse-seq prior to AP1 binding to show whether the binding site is nucleosome occupied prior to binding or not. Moreover, the data provided does not show that AP1 displaces a nucleosome in Fig. 4e. The reduction in the nucleosome bands upon AP1+SEP3 binding is fully attributable to the shifted band (arrow in Fig. 4e). Please remove this statement. Finally, Fig. 4e shows that AP1 cannot access its binding site in the context of the nucleosome at the AG locus; it can only bind when SEP3 is present. This should be discussed further.

***Reply:*** *Thanks for the suggestion. We remade the screenshots for Fig. 4b (now Fig.5d) to include newly generated MNAse-seq data prior to DEX induction to show the presence of nucleosomes in AP1 binding sites. As shown, the probes we used in Fig.4b (now Fig.5d) are occupied by nucleosomes before AP1 binding.*

*Thanks also for the reviewer’s interest and suggestions for Fig. 4e. In accordance with the reviewer comment, we decided to remove the statement and Fig. 4e from this manuscript, since this aligns well with our plans to further consolidate and investigate the in vitro mechanisms of AP1/SEP3 binding to nucleosomes in more depth.*

Additional comments: a. The Widom sequence is considered too strong a nucleosome positioning sequence by many in the pioneer TF field to assess in vitro binding of pioneer factors, it could be that SHL4/5 work well since they are close to the exit of the DNA from the nucleosome? Also the DBD can be placed in many other contexts on the nucleosome, I would think!

***Reply:*** *Thanks for the reviewer’s suggestion. We shifted the CArG boxes a few bps to make the A/T tract either in the concealed or exposed orientation at the Dyad and SHL-1-0 position, but the change of the orientation did not make AP1 bind to the dyad (Fig.S12). We also tested an additional SHL3-4 position and did not see a binding either (Fig.S12). What we repetitively see is the strong binding of AP1 to the SHL4-5 position, which is on the opposite side of the dyad and the A track is in the concealed position. Testing more positions and changing A tract length of the CArG box longer or shorter will be part of future research.*

b. Use of endogenous sequences is a plus here. However, in all cases the nucleosomes are not that well assembled (they should not have multiple bands). Did the authors us the heat step to isolate only stable nucleosomes for further experiments?

***Reply:*** *Thanks for the advice. Yes, we heated the nucleosomes at 37 °C and 42 °C, but this did not result in a more stable assembly. Heat treatment typically reduced all the forms (See* ***Figure*** *below). Only in the case of probe AP1 pro_3, 37°C treatment could dissociate unstable forms and left one strong band. After adding AP1 protein, we did see binding on the AP1 pro_3 nucleosome. In the case of SEP3 TSS, heat treatment removed all three forms of nucleosomes.*

Figure (for reviewer reply): Impact of different temperatures on nucleosome assembly at native genomic sequences (upper panel) and AP1 binding.

c. The free DNA bands should be included in the picture for Figs. 4d and e.

***Reply:*** *We have now clearly labelled the free probe bands of Fig. 5d (previously Fig. 4d). For reasons outlined above, Fig. 5e (previously Fig. 4e) has been removed from the manuscript.*

d. Throughout in all EMSAs it would be best to state the DNA and protein amount used and to calculate KDs, as described in Soufi Cell 2015.

***Reply:*** *We have now stated the DNA and protein concentration in the corresponding figure legends. We calculated the KDs for the naked DNA probes mentioned in point 1. We did not calculate KDs for the nucleosomes reconstituted with the native sequence as there are multiple bands for nucleosomes and multiple shifted bands after TF binding (Fig. 5d), which makes the calculation very complicated. It is also challenging to calculate the Kd for nucleosomes constituted with the Widom 601 sequence as the band of AP1-DNA complexes is the same size as nucleosomes (Fig. 5a).*

***REFERENCES***

*Barral A, Zaret KS. Pioneer factors: roles and their regulation in development. Trends Genet. 2024 Feb;40(2):134-148. doi: 10.1016/j.tig.2023.10.007.*

Kaufmann K, Wellmer F, Muiño JM, Ferrier T, Wuest SE, Kumar V, Serrano-Mislata A, Madueño F, Krajewski P, Meyerowitz EM, Angenent GC, Riechmann JL. Orchestration of floral initiation by APETALA1. Science. 2010 Apr 2;328(5974):85-9. doi: 10.1126/science.1185244.

*Lai X, Blanc-Mathieu R, GrandVuillemin L, Huang Y, Stigliani A, Lucas J, Thévenon E, Loue-Manifel J, Turchi L, Daher H, Brun-Hernandez E, Vachon G, Latrasse D, Benhamed M, Dumas R, Zubieta C, Parcy F. The LEAFY floral regulator displays pioneer transcription factor properties. Mol Plant. 2021 May 3;14(5):829-837. doi: 10.1016/j.molp.2021.03.004.*

*Pajoro A, Madrigal P, Muiño JM, Matus JT, Jin J, Mecchia MA, Debernardi JM, Palatnik JF, Balazadeh S, Arif M, Ó'Maoiléidigh DS, Wellmer F, Krajewski P, Riechmann JL, Angenent GC, Kaufmann K. Dynamics of chromatin accessibility and gene regulation by MADS-domain transcription factors in flower development. Genome Biol. 2014 Mar 3;15(3):R41. doi: 10.1186/gb-2014-15-3-r41.*

*Smaczniak C, Immink RG, Muiño JM, Blanvillain R, Busscher M, Busscher-Lange J, Dinh QD, Liu S, Westphal AH, Boeren S, Parcy F, Xu L, Carles CC, Angenent GC, Kaufmann K. Characterization of MADS-domain transcription factor complexes in Arabidopsis flower development. Proc Natl Acad Sci U S A. 2012 Jan 31;109(5):1560-5. doi: 10.1073/pnas.1112871109.*

*Wu MF, Sang Y, Bezhani S, Yamaguchi N, Han SK, Li Z, Su Y, Slewinski TL, Wagner D. SWI2/SNF2 chromatin remodeling ATPases overcome polycomb repression and control floral organ identity with the LEAFY and SEPALLATA3 transcription factors. Proc Natl Acad Sci U S A. 2012 Feb 28;109(9):3576-81. doi: 10.1073/pnas.1113409109.*

*Yan F, Powell DR, Curtis DJ, Wong NC. From reads to insight: a hitchhiker's guide to ATAC-seq data analysis. Genome Biol. 2020 Feb 3;21(1):22. doi: 10.1186/s13059-020-1929-3.*

**Second round of review**

**Reviewer 2**

The resubmitted manuscript by Xu and co-authors has adequately addressed my comments through performing additional in vitro experiments to strengthen their statement. I only have two minor comments.
1 The quality and labels of figures should be improved. For example, in Fig. 2c, "-" is missing for x and y axis.
2 In Fig. 4, "histone 3" should be "histone H3".

**Authors Response**

**Point-by-point responses to the reviewers’ comments:**

**Reviewer 2**

The resubmitted manuscript by Xu and co-authors has adequately addressed my comments through performing additional in vitro experiments to strengthen their statement. I only have two minor comments. 1 The quality and labels of figures should be improved. For example, in Fig. 2c, "-" is missing for x and y axis. 2 In Fig. 4, "histone 3" should be "histone H3".

*Reply: We thank the reviewer for calling our attention to these concerns. However, in regards to the first comment surrounding the labeling of Fig 2c, it appears that the appropriate “-“ was already in the original submission. We have changed histone 3 to histone H3 in Fig.4 as well as throughout the manuscript.*
